# Sensory Cortex Underpinnings of Traumatic Brain Injury Deficits

**DOI:** 10.1371/journal.pone.0052169

**Published:** 2012-12-21

**Authors:** Dasuni S. Alwis, Edwin B. Yan, Maria-Cristina Morganti-Kossmann, Ramesh Rajan

**Affiliations:** 1 Department of Physiology, Monash University, Clayton, Victoria, Australia; 2 National Trauma Research Institute, Alfred Hospital, Prahran, Victoria, Australia; Weill Cornell Medical College, United States of America

## Abstract

Traumatic brain injury (TBI) can result in persistent sensorimotor and cognitive deficits including long-term altered sensory processing. The few animal models of sensory cortical processing effects of TBI have been limited to examination of effects immediately after TBI and only in some layers of cortex. We have now used the rat whisker tactile system and the cortex processing whisker-derived input to provide a highly detailed description of TBI-induced long-term changes in neuronal responses across the entire columnar network in primary sensory cortex. Brain injury (n = 19) was induced using an impact acceleration method and sham controls received surgery only (n = 15). Animals were tested in a range of sensorimotor behaviour tasks prior to and up to 6 weeks post-injury when there were still significant sensorimotor behaviour deficits. At 8–10 weeks post-trauma, in terminal experiments, extracellular recordings were obtained from barrel cortex neurons in response to whisker motion, including motion that mimicked whisker motion observed in awake animals undertaking different tasks. In cortex, there were lamina-specific neuronal response alterations that appeared to reflect local circuit changes. Hyper-excitation was found only in supragranular layers involved in intra-areal processing and long-range integration, and only for stimulation with complex, naturalistic whisker motion patterns and not for stimulation with simple trapezoidal whisker motion. Thus TBI induces long-term directional changes in integrative sensory cortical layers that depend on the complexity of the incoming sensory information. The nature of these changes allow predictions as to what types of sensory processes may be affected in TBI and contribute to post-trauma sensorimotor deficits.

## Introduction

In many cases of brain disorders, sensory processing deficits may contribute significantly to the overall morbidity of the condition [1], [2], [3], [4], [5], [6], [7], [8], [9], [10], [11]. In the present study we examine the sensory cortical changes occurring in traumatic brain injury (TBI) which, in the US, affects approximately 1.7 million people annually, is a contributing factor in 30% of all injury-related deaths, and accounts for significant long-term hospitalizations across a range of populations [12].

Traumatic brain injury can occur from any blow to the head such as in car accidents, sporting field blows, and falls, and can produce very severe and life-long debilitating deficits in cognitive and sensorimotor function [13], [14]. In humans, persistent sensory deficits have been extensively demonstrated across a number of basic and complex sensory processing tasks [15], [16] with enhanced sensitivity reported in visual, auditory and touch processing in paediatric TBI patients for a year after injury [17] and auditory and visual deficits seen in adult TBI [18], [19]. The majority of TBI cases involve diffuse TBI resulting from acceleration/deceleration induced shear forces, where even MRI and CT scans show little or no visualizable damage suggesting that subtle alterations in neuronal function and circuit dynamics may underlie these deficits [20], [21], [22], [23].

This issue on the changes in neuronal processing of sensory input that could underlie diffuse TBI-induced disturbances in sensory behaviours has been examined in the rat barrel cortex that receives input from the mystacial whiskers that allow navigation in confined and complex spaces, permit detection of objects, and underlie object discrimination and recognition, with tactile acuity that easily matches that of humans [24]. Diffuse TBI results in prolonged heightened sensitivity to whisker stimulation in behaving rats [25] and, in barrel cortex, there is enhanced cFos activation with whisker stimulation [26] concomitant with the behaviour of tactile whisker hypersensitivity. There is a significant increase in glutamate neurotransmission in barrel cortex [27] without detectable cell loss [28], [29], [30]. However, anatomical markers of aberrant neuronal structure were present, suggesting that sensory morbidities could be attributed to axonal injury, a characteristic of diffuse TBI, and the secondary injury processes that render neurons in cortical and thalamic circuits susceptible to malfunction [21], [31]. In a recent study Ding et al. [32] used focal cortical compression to demonstrate that in the acute period after compression injury, following mechanical deformation and a transient loss of homeostasis, cortical responsiveness systematically increased over time (about 2 hours), likely through changes in the balance of excitation and inhibition. This appears to occur even without significant cell injury or death [32], consistent with other studies, mostly at the level of hippocampus, also showing hyperexcitability after trauma [33], [34], likely through changes in GABA receptors.

One limitation of these studies is that they were restricted to recording from layer 4 and also examined only effects in the short term (within 3 hours) after brain injury and therefore provide no information about dynamics of the full network over the long term nor the effects of the changes in excitation/inhibition balance on coding of whisker motions as used in natural behaviours. In fact this is a general limitation of the majority of studies investigating sensorimotor behaviour after diffuse TBI: they have only shown deficits over shorter time periods, have not focussed on sensory-specific morbidities, nor have they covered the dynamics of changes across the entire cortical network in a manner that can be related directly to normal sensory behaviour [35], [36], [37]. We now demonstrate that diffuse TBI leads to long-term hyper-excitation in response to sensory inputs, in supragranular barrel cortex layers but not in thalamorecipient layers (unlike the short-term effects described by Ding et al., 2011 [32]) or deep layers, and only to complex stimuli that model natural sensory behaviour. The changes in complex sensory encoding provide a basis for understanding the cortical origins of persistent sensory deficits in diffuse TBI as is needed for any understanding of how to manage or remediate the deficits beyond the immediate term after trauma.

## Materials and Methods

Adult male Sprague-Dawley rats (aged 10–12 weeks, 335–350 g) from the Alfred Medical Research and Education Precinct (AMREP) Animal Service were housed under a 12-h light/dark cycle with *ad libitum* food and water. Following 1 week acclimatisation, which included behaviour tests, animals underwent surgery to create diffuse traumatic brain injury (TBI) or for Sham controls. The cycle of acclimatisation, surgery, recovery and terminal electrophysiological experiments was done 5 times with 6–8 rats/cycle, allowing animals from a single cycle to be tested in terminal experiments in a short period in weeks 8–10 post-brain injury, removing any confound from age-related changes or from animals in a particular group being tested at different times post-surgery. In each cycle, littermates were randomly allocated to either TBI or Sham groups for surgical treatment.

### Ethics Statement

All experiments were conducted in accordance with the Code of Practice for the Care and Use of Animals for Scientific Purposes (National Health and Medical Research Council, Australia), and received approval from the AMREP Animal Ethics Committee and the Monash University Standing Committee on Ethics in Animal Experimentation (Approval numbers: E/1104/2011/M and SOBSA/P/2009/71, respectively).

### Induction of Traumatic Brain Injury

Brain injury was created in 19 rats using the weight-drop impact acceleration method [38], modified as previously described [35], while 14 littermates underwent sham surgery. Animals were initially anaesthetised with 5% isoflurane in 22% oxygen/78% nitrogen, intubated, and mechanically ventilated with 2–3% isoflurane in 22% oxygen/78% nitrogen. A metal disc (1 cm diameter and 3 mm thick) was fixed between bregma and lambda on the exposed skull with dental acrylic, the animal briefly disconnected from the ventilator to place it on a foam bed (Type E polyurethane foam, Foam2 Size, VA, USA) under the trauma device, and a 450 g weight dropped from 2 m through a vertical tube directly onto the metal disc [35]. Rats were then reattached to the ventilator until regular spontaneous breathing occurred, usually within 5 minutes, after which they were weaned off the ventilator. The metal disc was removed and the scalp incision sutured. Animals recovered with food and water provided *ad libitum*. Body temperature was maintained at 37.5°C using heat pads during surgery and for the next 24 h. Sham animals underwent the same surgical preparation as TBI animals but did not receive the traumatic impact.

### Behavioural Tests

Sensorimotor and cognitive function was assessed with a variety of tests, consisting of standard tests of TBI-induced deficits (rotarod, beam-walk and adhesive tape removal tests [36], and the Novel Object Recognition Test (NORT; [39])) and two whisker-based tests: the vibrissae-evoked forepaw placement test [40], [41] and specific components of the whisker nuisance task [25].

All animals were trained in rotarod, beam-walk and adhesive tape removal tests [36] in the week prior to surgery, with testing every second day. Post-surgery, tests were carried out from day 1, daily for 1 week, and then 1/week for the next 5 weeks. The rotarod test assesses sensorimotor coordination and grip strength by recording the maximum rotational speed at which animals can maintain their position on a motorised cylindrical rotating assembly of 18 stainless steel rods (Rateck, Australia). The beam balancing and walking task examined the ability to walk across a narrow beam suspended between two platforms 60 cm above the ground; normal walking for at least 0.5 m was scored as 0; crawling with the abdomen and tail touching the beam was scored as 1; inability to move on the beam was scored 2 and inability to balance on the beam was scored 3. The adhesive tape removal task, incorporating both sensory and precise motor function, measures the latency of adhesive tape removal from the forepaws. Each trial is terminated after 2 mins if the tape has not been removed. The number of tapes removed in this time was also recorded.

The vibrissae-evoked forepaw placement test was conducted once pre-surgery, then every second day in the week following surgery, and then 1/week for the next 6 weeks. Animals were supported along their trunk and hindquarters and brought close to a Plexiglas platform to allow contact by the whiskers to elicit a placing reaction in normal animals. A positive placing reaction was scored as 1 if it occurred within 5 seconds and delayed placing (>5 seconds) or failure to place within 5 seconds was scored as 0 [40], [41]. Each animal received 10 trials for each side of the body.

The second whisker-based test is a modified version of the whisker nuisance task used to examine persistent sensory deficits post-diffuse TBI [25]. This task consists of 8 behavioural categories and principal components analysis showed that the three most informative components differentiating between TBI and control animals were “response to stick”, “whisker position”, and “grooming”. Hence we selected these three components and tested once a week from weeks 2–6 post-trauma. Animals were placed in an empty cage for 5 minutes acclimatisation. The vibrissae on both sides of the face were then continuously stimulated for a period of 2 minutes using a wooden stick. Animals were scored on a 0–2 point non-parametric scale for whisker position, response to stick presentation, and grooming, with a score of 0 indicating the behavioural response was absent; 1 indicating that the response was present; and 2 indicating that the response was strong [25].

In week 7 post-surgery, the Novel Object Recognition Test (NORT) was conducted to examine exploratory behaviour, anxiety and memory function [36], [42]. In the test’s habituation phase, rats explored an empty enclosed arena for 15 min and were then briefly returned to their home cage. Then, in the learning phase two identical objects were placed in opposite corners of the arena before the animal was returned to explore for 15 min. After a 60 min time out in its home cage the rat was returned to the arena, where one familiar object had been replaced with a novel object, for a memory recall period of 15 minutes. The rat’s movements in the arena in all three phases were videotaped for analysis using a custom made automated movement recognition program. For the habituation period total body movement was calculated from this recording. For learning and recall phases object exploration/recognition was analysed as the time spent in an area 2 cm from the edge of the objects (measured as amount of time the rat’s nose entered this space).

### Methods for Extracellular Recordings from Barrel Cortex

In weeks 8–10 after surgery, TBI (n = 16) and Sham (n = 14) animals were tested in terminal experiments in which extracellular recordings were obtained from the posteromedial barrel subfield cortex (barrel cortex) using previously-detailed methods [43], [44]. Animals were anaesthetised with 5% halothane (Sigma-Aldrich, USA) and tracheotomised to maintain anaesthesia at 0.5–2% halothane through continuous mechanical ventilation (2.5–3.5 mL tidal volume, 72–80 breaths/min; both dependent on animal size). Anaesthesia depth was regularly monitored using continuous ECG/EMG recordings from needle electrode in the forepaw musculature, and regular checks of hindpaw, pinch and palpebral reflexes. Body temperature was maintained at 37–38°C by a heating pad thermostatically-controlled with feedback from a rectal probe (Fine Science Tools Inc., Canada).

Surgery [43], [44] was carried out to expose the skull and affix, via a screw and dental cement (Dentsply Intenational Inc., DE, USA), the head to a head bar, held in a stand, to hold the animal’s head firmly in place during surgery and recording. An area approximately 5 mm in diameter was drilled over the right barrel cortex (centred approx. 2.0 mm caudal to bregma; 6.0 mm lateral to the midline). The dura was then covered with a thin coating of silicone oil.

A parylene-coated tungsten microelectrode (2–4 MΩ; FHC, ME, U.S.A) was advanced, using an electronically controlled fast-stepping microdrive, through the dura to contact the cortical surface. Contact fidelity was confirmed through high-power microscopy and audiovisual monitoring of the amplified and filtered microelectrode output [43], [44]. The microdrive was zeroed at the surface and manual whisker deflection applied to determine if strong field potentials were obtained, confirming location over barrel cortex. Then the microelectrode was advanced in 5–10 µm steps, to a depth of 120–150 µm and halted in place to allow for any cortical dimpling to settle. Manual whisker deflections were applied to determine whether focal drive with a clear single Principal Whisker (PW; providing the main excitatory input) was obtained. Recordings were taken only in penetrations where good drive from a single PW was identified at 120–150 µm from the surface and at a depth in the granular (input) layer. If focal single PW drive was confirmed, the electrode was moved to a depth of 150 µm and stimuli applied to the PW. Thereafter recordings were collected every 100 µm, though not always sequentially in depth: to ensure that observations were not affected by any effects of anaesthesia over time, in some experiments recordings were taken first at deeper locations before the electrode was then moved up systematically to more superficial locations. At regular intervals in the penetration manual whisker deflections were used to confirm that the same PW was providing input, i.e., that the electrode was still within the same column.

The neural signal was amplified and band-pass filtered from 0.3–10 kHz and passed through a Schmitt trigger box which was used to set a voltage trigger level, approximately 1.5×>mean noise level at the recording location, for recording of cluster activity (see [43], [44]). Spike2 software was used to generate on-line displays of rasters of spike occurrences and peri-stimulus time histograms (PSTHs) during data collection [43], [44]. A copy of the filtered neural signal was also recorded by Spike2 at 83.33 kHz to allow offline extraction of single neuron data (detailed below).

### Controlled Whisker Deflections for Quantitative Barrel Cortex Data Collection

Computer-controlled PW deflections were applied to obtain data, using our novel motor-controlled lever arm system [43]. In essence, the whisker was threaded through a hole at the end of a lever arm on a motor assembly. The lever arm was moved along the whisker shaft to 5 mm from the mystacial pad. The whisker starting position for all recordings was standardised by moving the PW, using translators and goniometers in the assembly, so that it was perpendicular to the face. All recordings were obtained with the PW deflected from and returned to this rest position.

At each recording location a suite of 3 trapezoids with varied onset ramp velocity (60, 150 or 400 mm/s) with fixed deflection amplitude of 3.6 mm, ‘was applied to characterise cluster responses, and to extract single unit waveforms using spike sorting algorithms. Then, to record responses of barrel cortex to “naturalistic” whisker motion, suites of 4 complex motion waveforms were applied. During data collection, responses from the cluster were displayed online as PSTHs and raster displays [43], [44], while data from single neurons were displayed online as event times as they occurred; all data was stored for off-line analysis.

The 3 trapezoidal stimuli were generated de novo online using Spike 2 software and for on-line spike sorting, waveform templates were generated from responses to the suite of trapezoids, presented pseudo-randomly; 300–750 repetitions of each stimulus were used to obtain reliable waveforms for spike sorting. Standard Spike 2 template matching algorithms were applied to separate individual waveforms. A maximum of 6 waveforms (in most cases 3–4 waveforms) was obtained at any location. Throughout data collection the different single neuron waveforms were continuously monitored to ensure recording fidelity.

The 4 complex “naturalistic” whisker deflections were played out from text files which stored stimulus characteristics. These waveforms were generated from waveforms published in studies in which whisker motion had been imaged in awake behaving rats, and were those seen (a) in whisker motion across smooth and rough surfaces [45], (b) when rats made contact with a rod placed in the path of the whiskers [46], and (c) in head-fixed rats engaging in “free” whisking [47].

**Figure 1:**
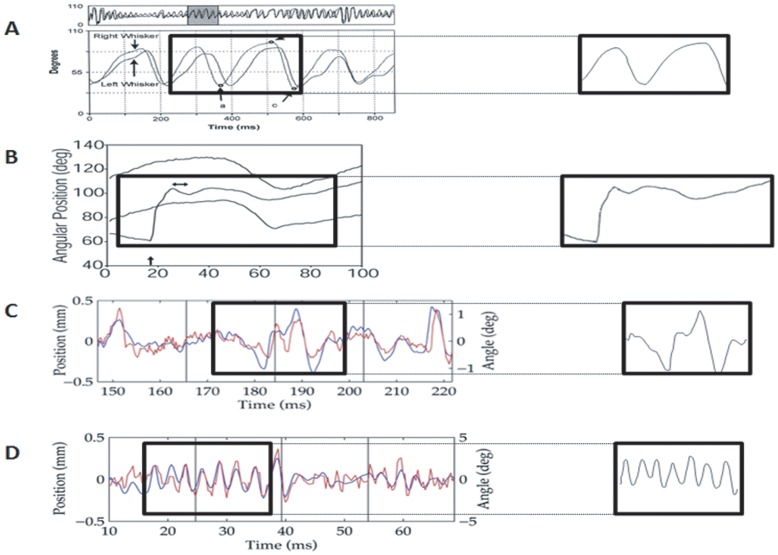
Four naturalistic stimulus waveforms used in extracellular recordings. (**A**) **Free whisking motion pattern.** Recorded video trace from an awake behaving rat performing exploratory whisking. Figure obtained from Gao et al ([47]; Figure 2). (**B**) **Object contact whisker motion pattern.** Recorded from an awake behaving rat moving a whisker to contact an object and brush past it. Figure obtained from Hartmann et al ([46]; Figure 8A). (**C**) **&** (**D**) **Rough and Smooth surface discriminatory whisker motion patterns, respectively.** Recorded video traces from awake behaving rats trained to discriminate between rough and smooth surfaces. Figures obtained from Ritt et al ([45]; Figures 3B & 5C, respectively).

These “naturalistic” complex whisker deflections were obtained from high-resolution images in the online versions of the three reports [45]–[47]. The full waveforms reported in the publications and the portion of the waveforms extracted to apply in our tests is shown in Figure 1. Except in the case of the “object contact” waveform [46], we did not apply the entire motion waveform reported but extracted a dominant feature of the waveform for use. For this, we imported the entire image in the appropriate online figure (see Fig. 1 legend for details) into an in-house MATLAB image processing program to define the image area-of-interest. The image was then scanned for the most intense pixel within the defined area and an output signal generated with the timing and amplitude of each detected pixel. An FIR filter was applied to smooth out any noise, and the signal re-sampled as close as possible to 100 kHz. Another low-pass FIR filter was applied to further smooth any high frequency jitter in the output graph, with the amount of filtering dependent on the quality, resolution, and line thickness of the original image.

The values of the movement axis of these waveforms were converted (generally from angular position in degrees) to distance in mm, with whisker position values normalised and scaled (without clipping the waveform) to a maximum deflection amplitude of 3.6 mm. These values were saved into a “signal” textfile of whisker position values (in mm) over time. A second ‘Stimulus Textfile’ was generated to determine how the signal textfile would be played out, including scaling factors to ensure the maximum amplitude did not exceed 3.6 mm. Spike2 software was used to open and play out the signal textfile, through the CED Power 1401 A–D converter to the motor controlling the lever arm that moved the whisker. As described before [43], the lever arm incorporated an optical sensor to monitor the actual displacement in real time and display this motion on the computer screen in synchrony with the neural data, to continuously ensure that the waveforms being generated by the text files were being reproduced faithfully by the stimulating motor and that the whiskers were moved in the desired pattern of motion.

The whisker motion waveform encoded in a signal textfile was played out as a suite of motions varying only in overall amplitude. Ten stimulus amplitudes were used in each suite, starting with 0.2 mm and then continuing from 0.4–3.6 mm, in 0.4 mm steps. Each amplitude was presented 50 times, with the different amplitudes in pseudo-random order across successive presentations.

### Quantifying Stimulus Features in the Naturalistic Whisker Deflections

The four “naturalistic” whisker deflections varied in waveform complexity. Due to the brevity of each stimulus, and the limited sample size (a single sample of each of the four complex whisker motion patterns from the original publications), standard spectral waveform analyses could not be applied to classify the stimuli. An alternative method of quantifying such complex waveforms is in terms of their fractal dimensions (FD; [48]). However, FD analysis requires a large sample size of waveforms and numerous repeats of each waveform. However analysis of short epoch self-affine waveforms (such as the whisker motions we used) is possible using the Normalized Length Density method [49] proposed for small-sample signals. Given a larger signal and sample size, the NLD value can also be converted [49] to the more commonly-used Higuchi FD for quantifying complexity of large-sample signals, through the construction of a calibration curve based on multiple repeats of each sample. However, the construction of an accurate calibration curve for the transformation of NLD to FD for signals specific to rat vibrissae motion is likely not possible due to the low number of signals available and a lack of knowledge of their actual FD, a statistic more accurately estimated for longer period signals which tend to repeat such as EEG and ECG [50]. Note that it is possible to rank the complexity of such short self-affine waveforms using NLD measurements without conversion to, and associated errors in, FD, simply because as NLD increases, FD also increases (although not linearly), and NLD is easily calculable for these types of waveforms [49].

### Data Analysis

#### Behaviour tests

Data were collected from rotarod, beam-walk, adhesive tape removal tests, whisker evoked forepaw placing test and whisker nuisance task over a period of 6–7 weeks which included pre- and post-trauma testing periods. Rotarod data were analysed using two-way repeated measures ANOVA. To obtain % of maximum rotarod, all scores were divided by the highest score each animal received in its pre-trauma/surgery tests. Beam walk, adhesive tape removal latency, whisker nuisance task and whisker evoked forepaw placing tests were analysed using a non-parametric Mann-Whitney U test. A p value of <0.05 was considered to be statistically significant. The novel object recognition test was analysed using a one-sample t-test with a hypothetical mean of 0.5 to determine statistical significance.

#### Electrophysiological recordings

Clusters and single neurons were segregated into lamina by depth as Layer 2 (150–300 µm from the cortical surface); Upper Layer 3 (350–500 µm); Deep Layer 3 (550–700 µm); Layer 4 (750–1000 µm); and Layer 5 (1100–1400 µm). A 50 µm buffer was used between layers to avoid allocating neurons to an incorrect layer. In practise we did not collect data from depths below 1500 µm from the cortical surface and so the Layer 5 data is likely to be almost totally from layer 5A only.

For all electrophysiological recordings data were collated as firing rate (in spikes/sec) in 1 msec bins over the period from 200 msec prior to stimulus onset until 100 msec post stimulus offset. Data from the clusters was used for offline analysis to generate population PSTHs to show the pattern of population responses within a lamina. For this, data for each stimulus (each of the 10 amplitudes for complex naturalistic stimulus waveforms, or each of three onset ramp velocities for the trapezoids) from each cluster in a lamina were averaged across all presentations of that stimulus. These average PSTHs were corrected for the pre-stimulus spontaneous activity using the 200 ms pre-stimulus firing rate, also averaged across all repetitions of the stimulus. A 5-point weighted moving average was applied to smooth out any noise and the data were then averaged across all multi-units to produce a grand PSTH. These grand PSTHs were used to identify the pattern of population responses to each stimulus and then determine appropriate counting windows for quantitative analyses.

For quantitative analysis for each stimulus waveform (each of the four complex naturalistic waveforms or the three trapezoids), we used only data from single neurons deemed to be responsive to that stimulus. A cell was deemed to be responsive if there were statistically significant responses at two or more consecutive stimulus *velocities* from the 3 test velocities used in the trapezoidal stimulus or *amplitudes* from the 10 test amplitudes used for each of the 4 naturalistic stimulus waveforms. We then extracted the peak firing rate, average firing rate and area under the curve for each stimulus. For these calculations, specific counting windows were established for each stimulus to encompass the maximum response over the stimulus presentation period. For the trapezoidal stimuli, a counting window from 5–50 ms after stimulus onset was used. The texture discrimination stimuli (rough and smooth) and object contact stimulus were analysed using a 5–30 ms counting window, while the free whisking stimulus was analysed using a 5–200 ms counting window.

Statistical analyses were carried out for each of the firing rate and temporal measurements for each stimulus waveform. For each set of results from a specific waveform, essentially two levels of analyses were carried out. The first was an omnibus mixed-model repeated measures ANOVA, consisting of the following factors: 2 Groups×5 Layers×10 Amplitudes (in the case of trapezoidal stimuli, 3 Velocities replaced the Amplitude factor). When significances were seen in this global analysis, repeated-measures ANOVAs were conducted for intra-laminar comparisons between the TBI and Sham groups. For this repeated-measures analysis the following factors were used: 2 Groups×10 Amplitudes (or 3 Velocities for the trapezoidal stimuli). Given this entailed a large number of within- and between-subjects comparison metrics in these repeated-measures ANOVAs, for clarity of reading, for these analyses we present only the meaning of the analyses in the Results section, with detailed metrics for each analysis presented in the Supplementary Information section. A p value of <0.05 was considered to be statistically significant. All data are reported as mean ± SEM.

## Results

### Sensorimotor and Cognitive Assessments

**Figure 2:**
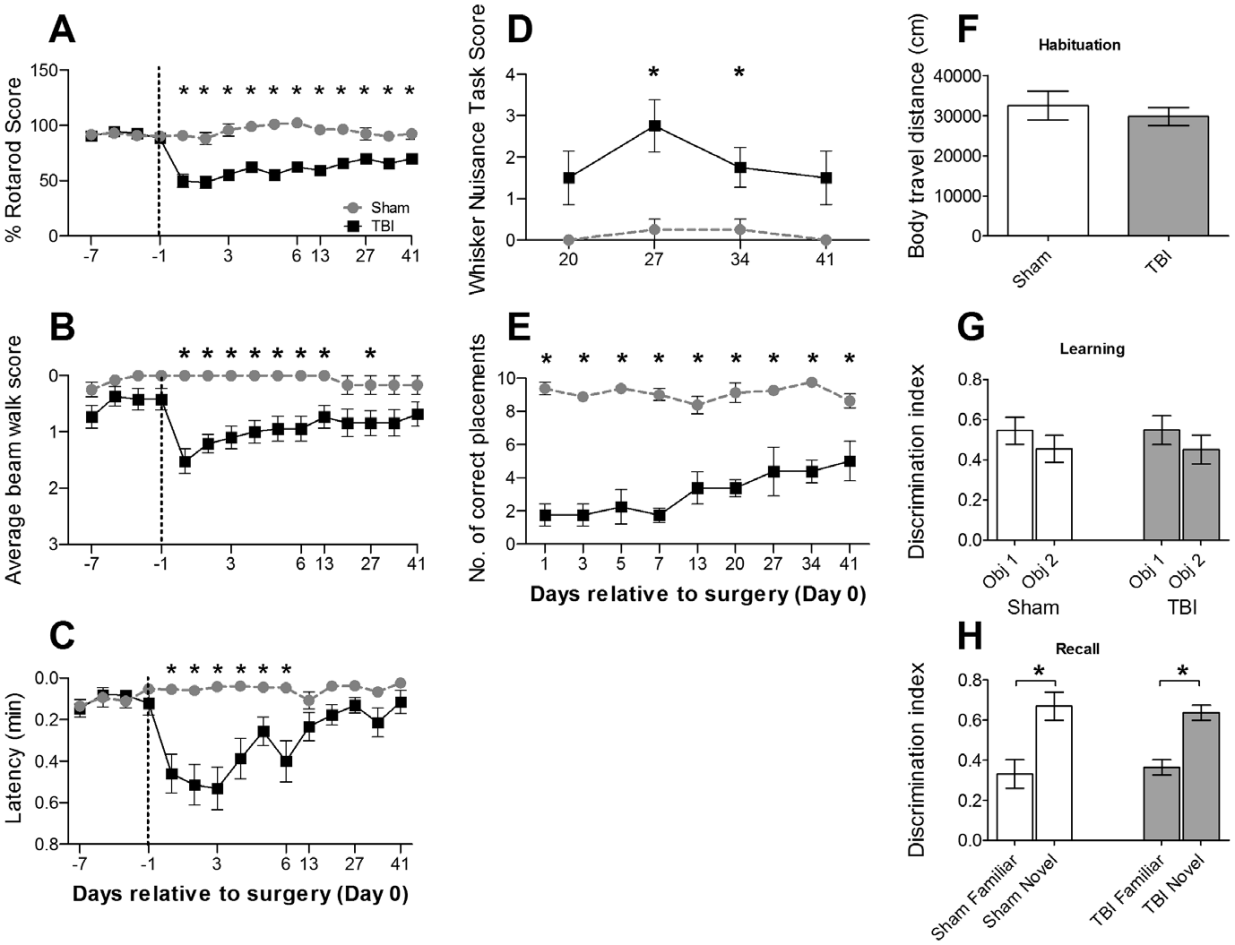
Sensori-motor deficits following diffuse traumatic brain injury (TBI). (**A**)–(**C**): **Changes in behaviour from prior to treatment and up to 6 weeks post- treatment.** In all panels the dotted line separates pre- (days −7 to −1) and post-surgery periods (days 1 to Week 6) for Sham (n = 12) and TBI (n = 19) groups. (**A**) **Grip strength and motor co-ordination:** Rotarod performance for each animal was expressed as a percentage of maximum pre-trauma rotarod score. This was averaged across all animals in Sham and TBI groups. (**B**) **Balance and motor co-ordination:** Beam-walking scores expressed as the average score for balance on the beam and ability to walk across it in Sham and TBI groups. (**C**) **Manual dexterity:** Adhesive tape removal latencies, expressed as average time in minutes to remove the first adhesive tape from the forepaws. (**D**)–(**E**): **Effects of impact brain injury on whisker-based sensori-motor tests.** Only post-treatment data were obtained for these tests in Sham (n = 4) and TBI (n = 4) groups. (**D**) **Whisker-evoked forepaw placement.** Correct forepaw placements (out of 10) averaged across left and right forepaws in Sham & TBI groups over 6 weeks post-trauma. (**E**) **Whisker nuisance task scores.** Total scores for Sham and TBI groups in the whisker position, response to stick presentation, and grooming components of whisker nuisance task. Scores were recorded from weeks 3 to 6. (**F–H**) **Novel Object recognition Test (NORT), of learning and memory.** The number of animals in the two groups was Sham n = 12 and TBI n = 19. (**F**) **Distance travel during habituation period.** Average distance (in centimetres) travelled. (**G**) **Discrimination index of object exploration during learning period.** Proportion of total time spent on exploring two identical objects (Obj 1 and 2). (**H**) **Discrimination index on exploring familiar and novel objects during recall trial.** Proportion of total time spent on exploring a familiar and novel object. * represents a significant difference of p<0.05. All values are mean ± SEM.

After trauma, TBI animals showed significant and persistent sensorimotor deficits (Fig. 2). For the three tests studied before and after injury there was no significant difference in performance between groups pre-trauma but on day 1 post-injury, TBI animals showed a significant decrease in rotarod scores (Fig. 2A; TBI = 49.8±5.8%; Sham = 90.8±4.1%; *p*<0.05), beam walk scores (Fig. 2B; TBI = 1.53±0.22; Sham = 0.0±0.0; *p*<0.05), and adhesive tape removal test of manual dexterity (Fig. 2C; TBI = 0.46±0.09 min; Sham = 0.06±0.01 min; *p*<0.05). Thereafter, TBI animals improved at different rates for the different tests. For the rotarod test, even at 6 weeks post-trauma they were significantly worse (TBI = 69.94±3.93%; Sham = 92.45±4.98%; *p*<0.05) while for the beam walk test significant deficits were present up to 6 days post-trauma (TBI = 0.95±0.22; Sham = 0.05±0.0; *p*<0.05), and at week 3 (TBI = 0.18±0.05; Sham = 0.04±0.0; *p*<0.05), with a gradual recovery such that from weeks 4 to 6 TBI animals were no longer worse than in Sham animals. For the adhesive tape removal test TBI animals improved over week 1 post-trauma, reaching a plateau 2 weeks post-trauma and thereafter showed no significant difference compared to Sham animals, consistent with previous work on effects in week 1 post-injury [51], [52].

Three other tests, all more directly linked to the whiskers, were applied only post-surgery. In the whisker-evoked forepaw placement test [41]; Fig. 2E), which relies on the animal’s ability to detect the presence of a surface using it’s whiskers alone, TBI animals demonstrated significant deficits in placing reactions from day 1 post-trauma (TBI = 1.75±0.66; Sham = 9.38±0.38; *p*<0.05) through to week 6 post-trauma (TBI = 5.00±1.1; Sham = 8.63±0.43; *p*<0.05). In a second whisker-based test, using particular components of the whisker nuisance task [25]; Fig. 2D), TBI animals showed more averse responses over the 4 week test period from weeks 3 to 6 post-trauma but this was significant only for weeks 3 (TBI = 1.5±0.65; Sham = 0.0±0.0; *p*<0.05), and 4 (TBI = 2.75±0.63; Sham = 0.25±0.25; *p*<0.05), likely due to the relatively small number of animals tested in this task, a late addition to our test battery. Finally, the novel object recognition test (NORT) conducted 7 weeks post-treatment showed no significant differences between TBI and Sham-surgery animals for all three NORT phases (Fig. 2F–H).

In general, across all behaviour tests, there were some persistent sensorimotor deficits in TBI animals even 6 weeks post-trauma. This was particularly evident in tasks more directly based on whisker sensory processing where TBI animals showed large, significant deficits in behaviour even 6 weeks post-treatment (Fig. 2D & E).

### Neuronal Responses to Complex Whisker Deflections

Electrophysiological data were obtained from all layers from 2 to 5 in TBI (n = 16) and Sham (n = 14) rats, with single cells spike-sorted from multi-neuron clusters, in response to four “naturalistic” whisker deflections and three trapezoidal deflections. A responsive unit (cluster or cell) was one with responses that were significantly greater, at *p* = 0.05, than their spontaneous rate (recorded in the 200 ms window prior to stimulus onset) over at least 3 successive stimulus amplitudes of the naturalistic stimuli and at least 2 successive trapezoid deflections. For each unit, the firing rate during the stimulus period was corrected for that unit’s spontaneous firing rate. Analysis of pre-stimulus spontaneous firing rates revealed no significant differences between groups.

For quantitative comparisons, we used the population grand PSTHs to identify different response components to each stimulus and to set appropriate counting windows to extract four metrics from all responsive single cells: (1) peak excitatory firing rate (PFR) in defined analysis window, (2) excitatory area under the curve of firing rate (EAUC) over the entire analysis window, (3) latency to the peak (L_PFR_), and (4) temporal dispersion of the peak response, measured as the duration in the analysis window over which firing rates were ≥50% of the PFR (T_1/2 PFR_). For all stimuli we found nearly identical effects for the two firing rate measures (PFR and EAUC), and nearly identical effects for the two timing measures (L_PFR_ and T_1/2 PFR_); hence we present data only from the PFR and the latency to the peak (L_PFR_).

#### (a) Object contact stimulus

**Figure 3:**
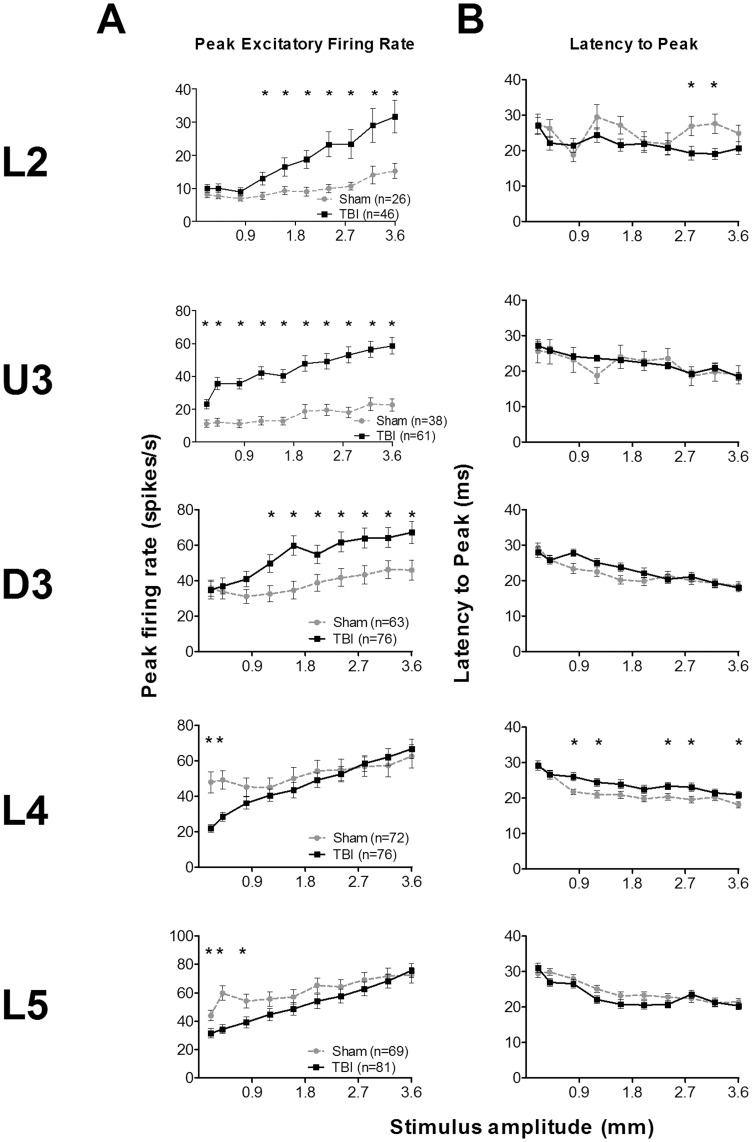
Effects of traumatic brain injury on responses to the “object contact” whisker motion waveform. Firing rate responses to stimulus applied to the Principal Whisker of single cells. For all columns the lamina from which data were obtained for each row is indicated on the left. (**A**)**,** (**B**) **Firing rate and temporal measures from responsive single cells for the onset response.** Single cell numbers for each layer are presented with the figure key. (*) represents a significant difference of p<0.05. All values are mean ± SEM. (**A**) **Peak Excitatory Firing Rate.** Data from single cells responsive to the stimulus (firing rates >pre-stimulus spontaneous activity) were analysed in the onset response window (shaded box in Fig. 3A) to determine the peak excitatory firing rate (PFR) of each cell at each stimulus amplitude (abscissa). (**B**) **Latency to peak (L_PFR_).** Time from stimulus onset to peak firing rate.

The object contact stimulus waveform modelled the whisker motion reported by Hartmann et al. [46] (refer to Fig. 8A from the same paper) in an unrestrained rat brushing its whiskers past a metal post to obtain a liquid reward (see Fig. 1 for stimulus waveform). Recordings were obtained from 77 responsive multi-unit clusters and 268 responsive single cells in Sham surgery animals and 76 responsive multi-unit clusters and 340 responsive single cells in TBI animals. There was no significant difference between the two groups in numbers of responsive clusters or cells in each layer (Clusters χ^2^ = 0.7, df = 4, *p>0.5*; Cells χ^2^ = 4.7, df = 4, *p>0.25*; number of clusters and cells per layer shown in Fig. 3A & B, respectively). This argues against any major difference in the two conditions in the ability to extract responsive cells in the different layers.

Grand PSTHs were generated by averaging the spike counts from all responsive multi-unit clusters and the Grand PSTHs obtained at the highest stimulus amplitude are shown in Figure S1 (described in Results S1). As shown there, this stimulus generated a very strong onset response occurring in the first 50 msec from stimulus onset and a strong offset response after the end of the stimulus (Fig. S1). We therefore used an analysis window of 5–50 ms from stimulus onset to extract single cell metrics for the excitatory onset response (shaded box, Fig. S1), and an analysis window from 70–130 msec post stimulus onset (dashed box, Fig. S1) for the offset response. Identical effects were seen for both components and we present only the outcomes of analyses for the onset response component.

#### Onset excitatory component: response metrics for single cells

As noted in Methods, for each set of results from a specific waveform, two sets of analyses were conducted: first, a global mixed-model repeated-measures ANOVA examining group, layer and amplitude/velocity effects and then laminar-specific repeated-measures ANOVAs examining group and amplitude/velocity effects. For clarity of reading, we present here only the meaning of the analyses, with detailed metrics for each analysis available in the Supplementary Information section.

Group data for the peak excitatory firing rate for the onset component (PFR_on_) are presented in Figure 3A. Mixed-model repeated measures ANOVA confirmed that TBI-induced changes varied with lamina. There were significant main effects of Group, Layer and Amplitude, with interdependency between pairs of factors but not a three way interaction (see Table S1 for significances). Given the layer-dependency of effects, 2-way repeated measures ANOVAs were used to analyse PFR_on_ data from each lamina. In all layers PFR_on_ increased systematically with stimulus amplitude (Fig. 3A). In all supragranular layers (L2, U3 and D3), PFR_on_ was generally greater in TBI cells (see Table S1 for significances). In contrast, there were generally non-significant differences between the two groups in L4 and L5; significant interactions between Group and Amplitude in these layers reflected significant differences between the groups for the lowest two (L4) or the three (L5) amplitudes – however, unlike in the supragranular layers, here TBI animals had *lower* PFR_on_.

We examined if the changes in response strength were accompanied by changes in timing, using the latency from stimulus onset to the onset peak (L_PFR_; Fig. 3B). Mixed-model repeated measures ANOVA found a generally systematic decrease in latency with increasing stimulus amplitude, with a main effect of Amplitude but no differences between groups or layers, nor an interaction between these terms (See Table S1 for significances). Laminar-specific analyses with 2-way repeated-measures ANOVA found always a significant systematic decrease in L_PFR_ with amplitude in all layers. For L2, there was a significant overall group difference (see Table S1) but no interaction, reflecting that at two higher amplitudes, TBI cases had faster L_PFR_. However, in the other supragranular layers, U3 and D3, there were no differences between groups. In L4 L_PFR_ was always *slower* in TBI cases. Finally, in L5, L_PFR_ was not significantly different between the two groups. In general, there were minor non-systematic changes in timing of the onset peak in the two groups except in the thalamic input layer 4, where L_PFR_ was always slower in TBI cases.

In general, there were only small, irregular changes in timing in TBI cases unlike the systematic effects seen with firing rate.

#### (b) Surface texture discrimination waveforms

**Figure 4:**
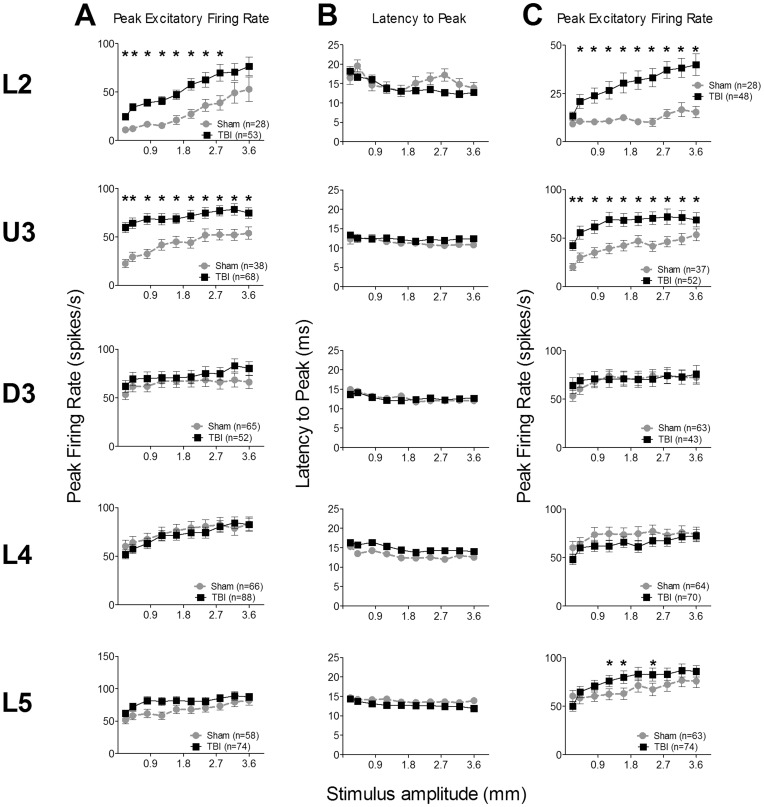
Effects of traumatic brain injury on responses to texture discriminative whisker motion waveforms. Firing rate and temporal metrics for responses from single cells to a “smooth surface” stimulus waveform (A, B) or a “rough surface” stimulus waveform (C) applied to their Principal Whisker (see Fig. 1 for stimuli waveforms). Data were obtained from single cells responsive to the stimuli (firing rates >pre-stimulus spontaneous activity) in the onset response window (5–30 ms post-stimulus onset in both cases), as a function of stimulus amplitude; the panels plot mean ± SEM. (*) represents a significant difference of p<0.05. The lamina from which data were obtained for each row is indicated to the left of the figure panels; cell numbers for each layer are listed in the key in the first column. (**A**) **Peak Excitatory Firing Rate to the “smooth surface” stimulus waveform.** Peak firing rate (PFR) in the in 5–30 ms post-stimulus onset analysis window. (**B**) **Latency to Peak Excitatory Firing Rate (L_PFR_) to the “smooth surface” stimulus waveform.** (**C**) **Peak Excitatory Firing Rate to the “rough surface” stimulus waveform.**

Ritt et al. [45] trained rats to discriminate between two halves of a vertical surface with “rough” and “smooth” regions and videographed the trained rats as they sampled the surfaces. We modelled (see Fig. 1) a dominant feature of the waveforms videographed in their report for whisker movement over a smooth surface and a rough surface. Since identical effects were seen for both waveforms, we detail the effects only for the smooth surface waveform, but illustrate the data for both waveforms in Figure 4.

As shown in Figure 5 of Ritt et al. [45], a dominant feature of vibrissae movement across a smooth surface was the presence of oscillatory motions. We applied one sequence of these motions (see Fig. 1) as shown in that report at each of 10 amplitudes while recording from 255 responsive single cells in Sham surgery animals and 335 responsive single cells in TBI animals. There was a significant difference between the two groups in the number of cells in each layer (χ2 = 12.1, df = 4, p<0.025) but not when layers were collapsed as supragranular (L2, U3 and D3), granular (L4) and infra-granular (L5) laminae (χ^2^ = 0.04, df = 2, p>0.95).

**Figure 5:**
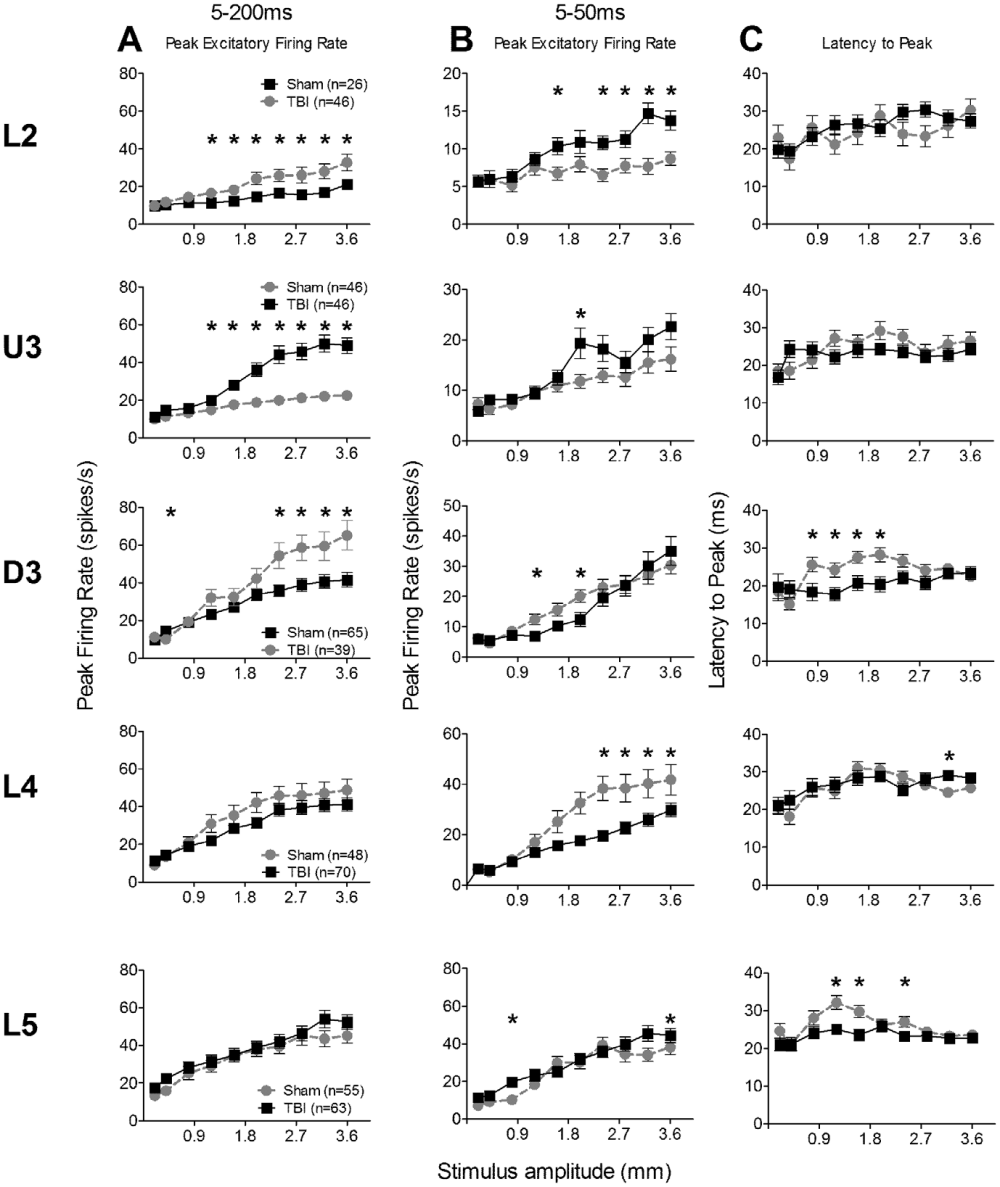
Effects of traumatic brain injury on single cell responses to the “free whisking” whisker motion waveform. Data from single cells responsive to the stimuli (firing rates >pre-stimulus spontaneous activity) measured in a response window (A: 5–200 ms post-stimulus onset) that encompassed the first full cycle of this two-wave stimulus (see Fig. 1) or a short onset response window similar to that used for other stimuli (B, C: 5–50 ms post-stimulus onset). (*) represents a significant difference of p<0.05. The panels plot mean data ± SEM; cell numbers for each dataset are presented in the key in column A; layers for each row indicated to left. (**A**) **Peak Excitatory Firing Rate (PFR) in 5–200 ms analysis window.** (**B**) **PFR in 5–50 ms window.** (**C**) **Latency to PFR (L_PFR_) in 5–50 ms window.**

The Grand PSTHs generated from all responsive multi-unit clusters to this stimulus (see Fig. S2; described in Results S1) showed that across all layers the dominant response element to was a single onset excitatory peak sometimes followed (especially in supra-granular layers) by a small second peak within (Sham L2) or outside (TBI D3) the stimulus period. Quantitative metrics were therefore obtained from single cells using an analysis window from 5–30 msec, encompassing the entire stimulus duration. For the peak firing rate (PFR; Fig. 4A) there was a significant effect of all main factors, with significant interactions between pairs of factors but not between all three (Table S2); i.e., firing rate varied between the groups, layers and amplitudes, and both group and amplitude effects depended on layer. Laminar-specific 2-way repeated-measures ANOVAs found that PFR always increased systematically and significantly with increasing stimulus amplitude. Straightforward group differences were seen in L2 and U3 where PFR was greater in TBI cells than in Sham surgery cells at most or all amplitudes. In all other layers there was no significant difference between the two conditions (Table S2).

For the timing measure (L_PFR_; Fig. 4B) too, any difference between groups was scattered between L2 and L4, with L2 showing only a significant Amplitude ×Group interaction while in L4 there was a significant Group effect but no interaction, with longer latencies in TBI cases. In all other layers there were no significant effects of Group or between Group and Amplitude (Table S2).

As noted, we also applied (Fig. 1) a dominant feature of the waveforms videographed by Ritt et al (2008; Fig. 3) in discrimination-trained rats as the vibrissae were moved over a “rough”’ surface. Based on the grand PSTHs (see below) an analysis window from 5–30 msec, encompassing the entire stimulus, was used to extract the standard quantitative metrics from single cells. The effects for PFR for this waveform (Fig. 4C) were similar to effects seen with the smooth surface discrimination motion waveform: straightforward across-amplitude Group differences were seen in L2 and U3, with no significant differences between the groups in the next three layers, D3, L4 and L5 (Table S3). In general, the effects in layers from D3-L5 were very similar for either discrimination motion waveforms with limited or no significant difference between the two groups. Similar effects were seen for the latency measure to this waveform (not illustrated) as for the other discrimination waveform.

Thus, in summary, for both discrimination waveforms, there were significant differences between the Sham surgery and TBI animals for the peak firing rate in cells in the top two supragranular layers. In all other layers there were no differences between groups. Latency to the peak response appeared uncorrelated to the PFR itself.

#### (c) Exploratory “free whisking” stimulus

The final naturalistic waveform we consider is that used to model the natural exploratory whisking in which rats move their whiskers freely to detect objects around them. This waveform was derived from recordings of whisker motion in head-restrained rats moving their whiskers freely in air [47] (see Fig. 1). Recordings were obtained from 240 responsive single cells in Sham surgery animals and 264 responsive single cells in TBI animals. There was a highly significant difference in the laminar distribution in number of cells (χ^2^ = 15.6, df = 4, p<0.01) but the direction of this difference varied with lamina; when collapsed into supragranular, granular and infra-granular laminae, there was no significant difference between the two groups, (χ^2^ = 3.65, df = 2, p>0.1).

In both groups this stimulus generated a number of complex response components (see Fig. S3) that could be separated into two blocks, each aligned with one of the two cycles of the motion waveform (see Fig. 1). An analysis window of 5–200 msec from stimulus onset encompassing all responses to the first motion cycle of this stimulus (Fig. S3; described in Results S1) was used to obtain quantitative metrics on firing rate from single cells. For the PFR (Fig. 5A), an omnibus mixed-model repeated measures ANOVA found a significant effect of all main factors and significant interactions between all factors (Table S4). Laminar-specific 2-way repeated measures ANOVAs showed that differences between groups were found only in supragranular layers (Fig. 5A; Table S4). Straightforward effects were seen in L2 where PFR was always greater in TBI cells than in Sham surgery cells (significant Group effect no significant interaction between Group and Amplitude). In U3 and D3, at lower motion amplitudes there were no differences in PFR between TBI and Sham surgery cells, but at all higher amplitudes PFR was always greater in TBI cells (significant Group and interaction effects). Finally, in L4 and L5 there were no significant differences between the groups (Table S4).

For measurements of timing we applied an analysis window from 5–50 msec post-stimulus onset which included only the first peak of responses to the first cycle of this stimulus (Fig. S3). This window is identical to that used for the “object contact” motion waveform and comparable to the 5–30 msec analysis window used for the two discrimination motion waveforms. Analysis of data for the latency to the peak, L_PFR_, from each lamina (Fig. 5C; Table S5) using 2-way ANOVAs showed that in D3 and L5 there were longer latencies in Sham cells at intermediate amplitudes, when compared with TBI cells, but in the other three layers there was no difference between the groups.

Thus, in general, for this stimulus, there was a firing rate difference between the two groups only in L2 and timing differences essentially only in L2 and U3, the top two supragranular layers.

#### (d) Inhibitory responses

**Figure 6:**
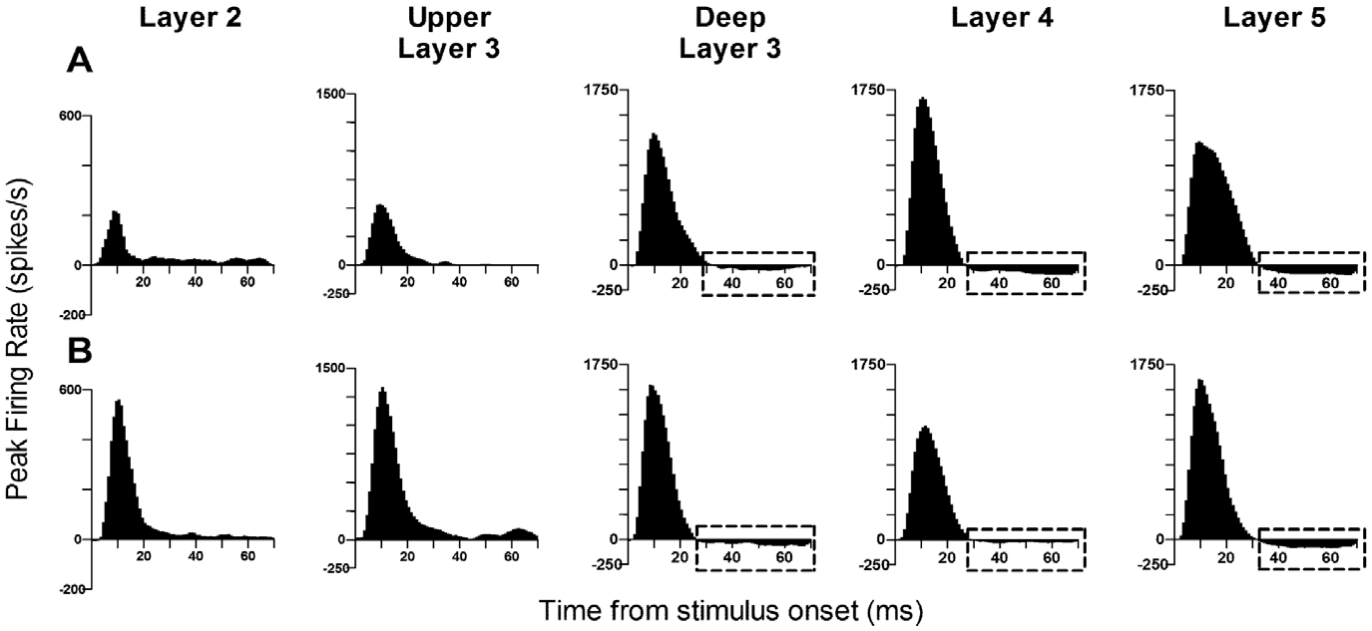
Effects of traumatic brain injury on inhibition in population responses. Population Grand peri-stimulus time histograms (PSTHs) of firing rate in Sham surgery animals (A) or TBI animals (B) to the “rough surface discrimination” whisker motion waveform. Firing rates data in each cluster was corrected for spontaneous firing rates (SFR) measured in the 200 msec period prior to stimulus onset and then averaged across all responsive clusters (clusters with peri-stimulus firing rates >pre-stimulus spontaneous activity) to generate layer-specific population Grand PSTHs for the stimulus applied at the largest amplitude (3.6 mm) to the PW. Boxes show inhibition in the population responses, where firing rate dropped below SFR.

TBI-induced hyper-excitation occurred in the presence of stimulus-driven inhibition. Figure 6 plots the pattern of responses (the population Grand Peri-stimulus time histograms of firing rate versus stimulus time; described in Results S1) to the “rough surface discrimination” waveform in the multi-unit clusters from which we spike-sorted the single cells data as presented above. These population responses show the effect reported above in single cells for this stimulus that, compared to Sham surgery cases, TBI cases show hyper-excitation in the upper two layers, minimal change in excitation in D3, a reduction in excitation in L4 and no apparent change in L5. It is noteworthy that in D3, L4 and L5 there was clear evidence of post-stimulus inhibition, with firing rates dropping below spontaneous firing rates in the 200 msec period prior to stimulus onset. Almost every naturalistic stimulus generated such inhibitory responses (responses below spontaneous activity) as can be seen in the grand PSTHs (Supplementary Figures S1–S3): for the first three naturalistic waveforms applied here, this effect always occurs in Layer 5 and occasionally in Layer 4 after the end of the onset response. The significance of this stimulus-evoked inhibition, as with others of the complex naturalistic stimuli detailed above, is discussed later.

### Neuronal Responses to Simple Whisker Deflections

#### (a) Trapezoidal whisker displacements

An important stimulus parameter affecting barrel cortex responses is velocity of whisker protraction, often studied by applying trapezoidal stimuli with varying onset-ramp velocities [44], [53], [54], [55]. At each recording location, we also applied such stimuli to the PW, using three trapezoids with varied onset ramp velocities of 60 mm/sec, a velocity which, in general, elicited weak-to-moderate responses in L4 in normal animals [44], 150 mm/sec which elicited strong but not saturated responses in L4 neurons [44], and 400 mm/sec at which most L4 neurons had saturated their firing rates [44]. Data were obtained from 244 responsive single cells in Sham surgery animals and 313 responsive single cells in TBI animals. In each layer, more cells were recorded from the TBI cases with a just-significant difference in the number of cells in each layer (χ^2^ = 9.7, df = 4, *p*<0.05); however, this significance was lost when the layers were collapsed into supragranular, granular and infra-granular laminae, (χ^2^ = 3.7, df = 2, *p*>0.1).

An analysis window of 5–50 ms from stimulus onset, set to cover the entire peak response (see Fig. S4; described in Results S1) at each of the three onset ramp velocities, was used to obtain quantitative metrics on firing rate.

**Figure 7:**
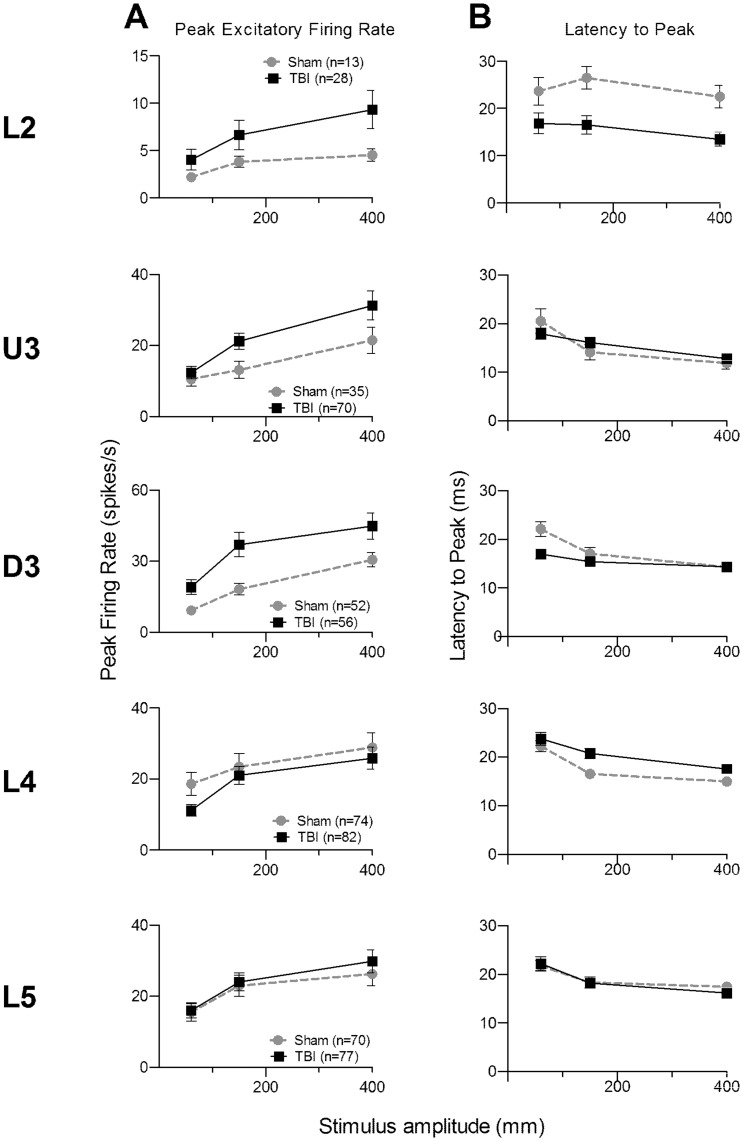
Effects of traumatic brain injury on single cell responses to trapezoidal whisker displacements. Data, from single cells responsive (firing rates >pre-stimulus spontaneous activity) to the stimulus applied to their PW (see Fig. 1 for stimuli waveforms), in the onset response window (5–50 ms post-stimulus onset); the panels plot mean ± SEM. Cell numbers for each layer are presented in the key in (A). (**A**) **Peak Excitatory Firing Rate (PFR).** (**B**) **Latency to PFR (L_PFR_).**

For the PFR (Fig. 7A) an omnibus mixed-model repeated measures ANOVA found a significant effect of all main factors and significant interactions between all pairs of factors, but not a three-way interaction (Table S6). Laminar-specific 2-way repeated measures ANOVAs found (Fig. 7A; Table S6) a significant difference in PFR between groups only in D3 where PFR at all three velocities was significantly greater in TBI animals than in Sham surgery animals. Laminar-specific data analysis of the latency to the peak response, L_PFR_ (Fig. 7B; Table S6), with 2-way ANOVAs showed that significant differences between groups occurred non-systematically, e.g., L2 cells in TBI animals showed shorter latencies at the two highest velocities, but L4 cells in Sham surgery animals showed shorter latencies at the two highest velocities. In D3 significantly shorter latencies were found in TBI cells but only for lowest velocity (see Fig. 7B). In U3 and L5 there were no differences between the groups. Thus, for this stimulus type, there was no significant TBI-induced change in responses.

#### (b) Ramp component of the exploratory whisking motion waveform as a simple stimulus

As shown above, the hyper-excitation in supragranular layers in TBI cells when using complex naturalistic stimuli was not revealed when trapezoid waveforms were applied. The trapezoid ramp velocities were selected to cover the range from one eliciting weak responses in most L4 cells in normal animals, through to one at which most L4 neurons had saturated firing rates [44]. Hence, the absence of clearly differentiated effects with these stimuli cannot be due to either ceiling or floor effects that masked TBI-induced changes. Instead it appeared that the TBI-induced changes required complex whisker motions to be revealed.

A negative test of this hypothesis was available using the exploratory whisking stimulus. The early part of this stimulus is essentially a ramp (see Fig. 1). In our testing we varied stimulus amplitude, using 10 amplitudes, and hence this early phase essentially represents a velocity ramp with different velocities across the 10 amplitudes. Analysis of the response to only this “ramp-like” component of the complex stimulus could allow examination of the hypothesis that a simple ramp-like stimulus, although clearly a strong driver of barrel cortex excitation, may not engage the complex neuronal interactions affected in TBI and therefore not differentiate barrel cortex responses in TBI from responses in Sham surgery animals. Hence we applied an analysis window of 5–50 msec to the exploratory whisking stimulus; as seen from Figure 1, this would span only the onset ramp of the first cycle of the stimulus.

Metrics on firing rate were obtained in the 5–50 ms analysis window. For the PFR (Fig. 5B) an omnibus mixed-model repeated measures ANOVA found no significant effect of Group but a significant effect of Amplitude and Layer and significant interactions between some factors (Group × Layer; Amplitude × Layer; Amplitude × Group × Layer), and but not others (Amplitude ×Group; see Table S5 for significances). The PFR data from each lamina were analysed separately using 2-way repeated measures ANOVAs. Only L2 and L4 showed any differences between groups but only at the higher amplitudes and the direction of effects was opposed in the groups: TBI cells had stronger responses in L2 and Sham surgery cells had stronger responses in L4. In U3, D3 and L5 there was no significant Group effect and there was a significant Group x Amplitude interaction only in U3.

Overall, use of a shorter analysis window, restricted to the onset response evoked by a ramp-like component of the stimulus, showed different effects to those seen with an analysis window over the full first cycle of the stimulus. With the shorter window, a significant increase in excitation in TBI cells was seen only in L2 whereas with the longer window, an increase in excitation in TBI cells was seen in all supragranular layers. However, with the shorter window there was a significant decrease in responses in TBI cells in L4 compared to Sham surgery cells; with the longer analysis window, a decrease in responses in TBI cells in L4 compared to Sham surgery cells was found but was insignificant. The more-restricted laminar differences when analysis of responses to a complex stimulus was done with the shorter window provide a measure of support for the hypothesis that when a stimulus waveform is relatively simple, it may not engage the full range of neuronal interactions seen with more complex waveforms.

### Summary of Effects of TBI

**Figure 8:**
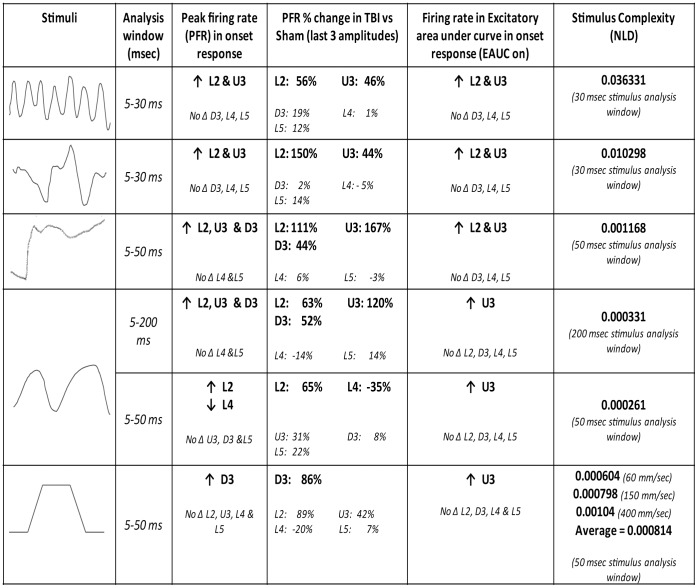
Summary of key effects of TBI on single cells for each whisker motion stimulus. The stimuli (column 1; see text for details) are arranged from top to bottom in order of NLD value (column 6: a measure of stimulus complexity) for the stimulus during the analysis window (column 2) from which data were extracted from single cells. The stimulus waveforms were, in succession from top to bottom, for smooth surface discrimination [45], rough surface discrimination [45], object contact [46], exploratory free whisking [47] and trapezoids. Columns 3–5 summarize the key cortical changes (highlighted in bold): the two firing rate measures, peak firing rate in the analysis window (columns 3 and 4) or excitatory area under the firing rate plot over the analysis window (column 5); columns 3 and 5 present a summary of single cell effects described in the text while column 4 presents the percentage change in mean PFR value across all TBI cells compared to mean PFR value across all Sham surgery cells (significant changes in bold).

Figure 8 summarises our key findings for the two firing rate measures, PFR and the EAUC, since there were rarely any changes in timing measures. For all complex, naturalistic whisker motion stimuli, neuronal excitability increased but only in supragranular layers of the TBI barrel cortex (Column 4). This effect was found when the analysis window covered the stimulus duration, across very different stimulus durations (Column 2). Most consistently across both measures, the increase in excitation in TBI was in Layers 2 and Upper 3; PFR could also be higher in Deep 3 in TBI animals. Finally, there were rarely any changes in the input (Layer 4) and output (Layer 5) layers of cortex in either firing rate measures or timing latency measures after diffuse TBI.

Significant changes in responses in supragranular layers were not seen with simple trapezoid stimuli and this could be partly replicated with a complex stimulus (the exploratory whisking stimulus) when analysis was restricted to only the ramp component of that stimulus. This was not due to using a narrower analysis window since significant supragranular changes in TBI were seen with similar or shorter analyses windows applied to the other three naturalistic stimuli. We explored the issue of stimulus complexity in these effects by calculating the NLD value as an index of stimulus complexity (see Methods). There was some degree of correspondence between stimulus complexity and changes in PFR but the changes could not be completely explained by stimulus complexity per se since trapezoidal stimuli, which did not reveal the supragranular hyper-excitation, had an average NLD value greater than that for the full exploratory whisking stimulus. The results suggest that NLD analysis is the most appropriate measure to quantify the stimuli in terms of complexity, and that the NLD metric can be used to classify such short-duration naturalistic stimulus waveforms and at least partially differentiates cortical neuronal response diversity.

The Kalauzi et al. models [49] for conversion of NLD values to the fractal dimension (FD) lead to negative FD for the NLD values calculated for the four waveforms. Mandelbrot [48] has noted that FD >0 represented the fine-grained non-random multifractal (“thermodynamic”) properties of samples and FD <0 represented the *variability* between samples of coarse-grained (“mesopic”) multifractal properties. It seems likely then that the latter mesopic dimensions provide a better descriptor of the differences between the four signals which might allow us to understand the differences between barrel cortex neuronal responses evoked by these signals.

## Discussion

This is the first study to describe in any detail at all, the long-term consequences of diffuse TBI across the columnar network in primary sensory cortex, and amongst a small number of studies examining diffuse TBI more than 1–2 weeks post-trauma. Our results indicate that TBI induced hyper-excitation in firing rates but only in supragranular layers of barrel cortex; consistent with our conclusion that this occurred through cortex-specific changes, there were no changes in response timing as might be expected for relayed sub-cortical effects. As discussed below, our data indicate that a likely mechanism for the hyper-excitation is loss of one form of intra-cortical inhibition, surround inhibition.

### Mechanisms for Changes in Cortical Excitability

Given that this is, to the best of our knowledge, the only study that has described the changes across the entire columnar network after brain injury, especially in the longer-term, and to complex stimuli, it is difficult to find parallel studies that could provide some insights into the mechanisms. Ding et al. [32] found a global suppression of responses in Layer 4 from 5–20 minutes post-cortical compression, followed by increased activation above baseline 2 hours post-injury. This may superficially appear related to our effects in showing post-TBI effects in barrel cortex to consist of changes in the balance of excitation and inhibition [32]. However it is unlikely to have much bearing on our observations since these effects were studied only in a period of a few hours post-TBI, were examined only in layer 4, and only to simple paired-pulse stimuli and therefore provide no information about what happens across the full network, over the long term, or the consequences on coding of whisker motions as used in natural behaviours. In all these regards our study makes novel contributions.

Hall and Lifshitz [26] reported barrel cortex neuronal cFos activation to whisker stimulation showed rebound activity in cortex, thalamus and hippocampus from 28–42 days post-injury. Post-TBI hyperexcitability has been linked to increases in frequency and amplitude of spontaneous excitatory synaptic currents and a decrease in frequency of spontaneous inhibitory synaptic currents in Layer 5 at 2–6 weeks post-injury though not in supragranular layers [56]. However, the conclusion that TBI causes a shift towards excitation early after injury, which persists for many weeks, is not without debate [57], [58], [59], [60]. Sensory cortical response changes have also been suggested to occur through sub-cortical changes [28], [61] leading to increased cortical activation [62], [63]. However, the general absence of changes in layer 4 post-TBI in our study, and the absence of any timing changes in supragranular layers where hyper-excitation did occur, suggests that our long-term effects are unlikely due to changes in thalamic input directly or indirectly altering cortical interactions.

Increased excitation is commonly seen after other cortical injuries [64], likely from initial loss of inhibitory neurons [65] followed by secondary injury processes [66], [67] including down-regulation of the K^+^-Cl^-^ co-transporter 2 which maintains Cl^-^ concentrations [33], decreased expression of inhibitory post-synaptic receptors [68], or decreases in inhibitory synapses [69]. Decreased GABAergic inhibition plays an important role in epileptogenesis [70] and in ischemic stroke [71] and is confirmed by electrophysiological studies of stroke, showing increased cortical excitation [72].

Hyper-excitability in supragranular layers with little or no change in deeper layers may reflect a simple direct distance-dependent effect of damage from the impact trauma. Previous histological studies from our laboratory using this model [35], [36], [73] found damage mainly in the corpus callosum (CC) (e.g., [36]) and this was also found in those animals of the current study examined histologically at the 8 week post-injury time point, with some apparent cell loss in hippocampus but no cell damage in barrel cortex. This is consistent with work by Li et al [74] which shows similar histological evidence of damage in the CC but not extending past 2.2 µm from the midline suture, well medial of the barrel cortex. It is therefore unlikely that the supragranular hyper-excitability is solely due to direct impact effects of the trauma protocol.

Reductions in, rather than loss of, inhibition more likely account for long-term post-TBI hyper-excitability. Diffuse TBI does not typically result in overt lesions or cell death in cortex or thalamus but causes widespread damage to neuronal structure and cerebral vasculature [28], [38], [75]. In particular, white matter tracts of corpus callosum and brainstem suffer axonal swelling and injury [76], triggering astrocytosis and macrophage infiltration [35]. In Layer 5 of mouse neocortex, axon regeneration occurs 28 days post-TBI [77], causing circuit reorganisation and plasticity. In somatosensory cortex while there is no cell death, there is neuronal atrophy which spreads from upper and barrel layers at day 1 post-injury to middle and deep layers by day 7, and to deep layers, white matter and inter-barrel septa by day 28 [28], [75]. How this affects cortical inhibitory processes is unknown.

We propose that select loss of only one form of cortical inhibition may account for the effects we report. Auditory cortical inhibition is differentiated into surround and within-field inhibition (arising outside and within the neuron’s excitatory response area, respectively; c.f. [78] and only the former is affected in peripheral injury-induced cortical change [79]. Then, loss of surround inhibition resulted in stronger responses even to stimuli from within the response area, despite preservation of in-field inhibition [79]. This is consistent with our finding of hyper-excitation to stimuli to the principal whisker (i.e., to stimuli from within the response area) with preservation of in-field stimulus-driven inhibition (e.g., Fig. 6). In auditory cortex surround inhibition shapes responses predominantly to complex stimuli [78] and this may account for the fact that in the present study, hyper-excitation was more predominant to complex whisker movements.

### TBI Changes in Relation to Stimulus Features

To correlate stimulus and TBI-induced effects, we attempted to classify the naturalistic waveforms but, due to the short length of the stimuli and the signals’ self-affine natures, standard spectral and temporal analyses could not be used. We therefore examined whether specific stimulus features known to be important in activating responses in barrel cortex neurons, such as stimulus velocity or vibration frequency [24], [54], [80], could differentiate effects seen with the different stimuli. For example, differences in the velocity of the onset component of the naturalistic waveforms compared to the velocity range used in the ramps of the three trapezoids could be responsible for differences in TBI-induced effects on neuronal responses. That this is not the case can be seen by consideration of neuronal responses between TBI and control cases for the object contact motion versus the trapezoids. The complex whisker motion, which has a clear onset ramp feature, was tested at amplitudes from 0.2–3.6 mm and the velocity of the onset component varied systematically with amplitude, ranging from 60 mm/s to 1095 mm/s over the test amplitude range. For the three trapezoids, onset ramp velocities were 60, 150 and 400 mm/s which were well encompassed within the range of velocities of the onset component of the object contact stimulus for the 4 lowest stimulus amplitudes of 0.2, 0.4, 0.8 and 1.2 mm (61–365 mm/sec). Nevertheless there were clear differences between effects with the two types of stimuli: neuronal responses differed between TBI and Sham cases for at least some amplitudes of the complex stimulus but not for any of the three trapezoids. This a faster onset velocity per se in the complex stimuli is unlikely to be responsible for revealing TBI-induced hyper-excitation to these stimuli but not to the trapezoids.

A second possibility is that whisker vibration in the complex whisker motions (e.g., in the smooth surface discrimination waveform and exploratory free whisking motion) could have revealed TBI-induced neuronal hyper-excitation that was not revealed by the trapezoidal stimuli. However, TBI-induced supragranular neuronal hyper-excitation was seen with the rough surface discrimination waveform which did not have a vibratory component; further it has been well demonstrated that vibration frequency is most likely encoded by the velocity [54].

Thus, it is not the individual stimulus components but a combination of properties (stimulus *complexity*) that could explain the difference in effects to the naturalistic stimuli versus the trapezoids, and even perhaps between the naturalistic stimuli. The small number of samples (waveforms) and the absence of repeats of each sample precluded standard fractal dimension analysis but allowed use of the NLD value as a measure of stimulus complexity. The TBI-induced hyper-excitation generally occurred with stimuli with higher NLD values (see Fig. 8), consistent with the general notion that a combination of properties in the overall stimulus could explain the TBI-induced difference in response strength to the naturalistic stimuli.

### Behavioural Morbidities after TBI in Rats and Relationship to Effects in Humans

We found long-term motor deficits in rotarod, beam walk and adhesive tape latency tests applied post-TBI [81]. Significant reductions in rotarod scores up to 3 weeks post-injury have been reported before [82] but ours is the first study showing longer-term deficits. We also used the vibrissae-evoked forepaw placement test and the whisker nuisance task, to examine whisker-specific deficits. Both tests have been used to revealed deficits in TBI [25], [41], [83], and we found persistent deficits over 6 weeks post-injury. In the whisker nuisance task hypersensitivity to whisker stimulation peaked 4 weeks post-injury as also reported previously [25], [83].

Sensorimotor deficits could be due to peripheral changes [41], [81] or disruptions in sensorimotor processing networks [26]. Whisker-based sensory behavioural morbidity has been attributed to thalamic neuronal damage and atrophy [26], [28] but this shows recovery at one-month post-axotomy from re-establishment of trophic support, as indicated by expression of plasticity markers such as GAP-43 and synaptophysin in thalamus and hippocampus early after TBI [26], [84] and in retina after retinal axonal injury [85]. Persistence of behavioural morbidities over 6 weeks post-injury in our study suggests axonal repair over this time does not compensate for TBI-induced circuit changes or may even be maladaptive.

### Implications of Changes in Processing of Complex Sensory Stimuli but not Simple Sensory Stimuli

The core result of our study is that diffuse TBI causes supragranular hyper-excitability that alters the processing of complex naturalistic stimuli but not simpler stimuli, even when the latter contain a critical informative component known to activate barrel cortex neurons very strongly. Stimulus complexity-dependent changes in neuronal processing are seen in cases of human TBI where patients often show sensory deficits specific to processing of complex sensory cues [16], e.g., Brosseau-Lachaine et al [15] showed deficits in dynamic orientation-identification thresholds up to 12 weeks post- injury, while more simple static thresholds remained unaffected. Our results suggest that human studies using complex, naturalistic stimuli will better reveal the full extent of human sensory processing deficits post-TBI than simple threshold or detection stimuli, and this can be linked to supragranular sensory cortical changes.

Supragranular hyper-excitation may play some part in the human hypersensitive behavioural morbidities seen after brain injury [86], [87]. Sensory hypersensitivity in response to salient stimuli has also been reported in schizophrenia [6] and such hypersensitivity could correlate with the persistent sensory (whisker)-related morbidities we describe. Finally, aversion to sensory overstimulation is also seen in other neurologic disorders such as schizophrenia and Fragile X Syndrome where excitation/inhibition balances are thought to play a role [88], [89], [90], [91] and changes such as we describe may also apply in those cases.

In humans, persistent diffuse brain injury-related sensory deficits occurs across modalities [18], [19], [92], though often such studies were unable to separate sensory deficits from physical damage to the sensory organs or to central processing dysfunction. The impact/acceleration model of TBI ensures that damage to the sensory organ in question (the whisker system) does not occur. It has recently been suggested that sensory processes play a more primary role in the integration and functioning of higher order cognitive abilities [93], [94] supporting studies investigating the link between sensory deficits and the persistent cognitive dysfunction that is prevalent after brain injury [95], [96] to the point where testing of sensory systems after TBI is now considered to provide valuable information regarding cognitive processing and possible rehabilitation paradigms [96].

Finally, we note that the highly detailed description of the sensory encoding effect of TBI that we have found provides a basis for understanding what primary sensory cortical disturbances occur in TBI and likely underpin TBI-induced perceptual, motor and cognitive deficits. We found supra-granular hyper-excitation in the long-term after TBI accompanied by normal granular responses. Ding et al [32] found that within two hours of brain injury, granular layers (the only layer examined) showed hyper-excitation. This suggests that the changes we report are not the same as those reported immediately after brain injury but may evolve over time such that the granular layers recover (the fate of supra-granular layers immediately post-injury being unknown). It is therefore important to determine the temporal evolution of supra-granular hyper-excitation and recovery of granular normality, and our current work is focused on this. Additionally, we aim to explore how stimulus complexity can be a determining factor in revealing the neuronal encoding changes caused by brain injury. While the present study has not fully explored the neuronal mechanisms behind these sensory cortical disturbances, this data will guide future studies, in brain slice and other such preparations, of the mechanisms underlying sensory cortical changes in TBI, and in psychophysical studies, of the detailed sensory consequences of TBI.

## Supporting Information

Figure S1 **Effects of traumatic brain injury on pattern of responses to the “object contact” whisker motion waveform: Population Grand PSTHs to the largest stimulus waveform (3.6 mm total deflection).** Firing rates were averaged across all responsive clusters (firing rates >pre-stimulus spontaneous activity; cluster numbers listed in shaded region) in Sham surgery animals and in TBI animals. The first column of PSTHs is from the Sham surgery cases and the second column from the TBI cases. The boxed areas represent the analysis windows from 5–50 ms post-stimulus onset to capture the onset response (shaded box) and the window from 70–130 ms post- stimulus onset to capture the offset response (dashed box). For all columns the lamina from which data were obtained for each row is indicated to the left of the PSTHs. Cluster numbers for each lamina and group are also presented.
(TIF)

Figure S2 **Effects of traumatic brain injury on pattern of responses to the “smooth surface discrimination” whisker motion waveform: Population Grand PSTHs to the largest stimulus waveform (3.6 mm total deflection).** Firing rates were averaged across all responsive clusters (firing rates >pre-stimulus spontaneous activity; cluster numbers listed in shaded region) in Sham surgery animals and in TBI animals. The first column of PSTHs is from the Sham surgery cases and the second column from the TBI cases. The boxed areas represent the analysis window from 5–30 ms post-stimulus onset to capture the onset response. Figure conventions as for Figure S1.
(TIF)

Figure S3 **Effects of traumatic brain injury on pattern of responses to the “free whisking” whisker motion waveform: Population Grand PSTHs to the largest stimulus waveform (3.6 mm total deflection).** Firing rates were averaged across all responsive clusters (firing rates >pre-stimulus spontaneous activity; cluster numbers listed in shaded region) in Sham surgery animals and in TBI animals. The first column of PSTHs is from the Sham surgery cases and the second column from the TBI cases. The boxed areas represent the analysis windows from 5–50 ms post-stimulus onset to capture the onset response (dashed box) and the window from 5–200 ms post- stimulus onset to capture the first cycle of the stimulus (shaded box). Figure conventions as for Figure S1.
(TIF)

Figure S4 **Effects of traumatic brain injury on pattern of responses to the trapezoidal whisker motion waveform: Population Grand PSTHs to the highest stimulus velocity (400 m/s).** Firing rates were averaged across all responsive clusters (firing rates >pre-stimulus spontaneous activity; cluster numbers listed in shaded region) in Sham surgery animals and in TBI animals. The first column of PSTHs is from the Sham surgery cases and the second column from the TBI cases. The boxed areas represent the analysis window from 5–50 ms post-stimulus onset to capture the onset response. Figure conventions as for Figure S1.
(TIF)

Table S1 **Results of statistical analysis of firing rate (PFR_on_) and temporal measures (L_PFR_) in single cells responsive to the object contact whisker motion stimulus from 5–50** **ms from stimulus onset (related to** **Figure 3** **).** The Table lists F statistics and degrees of freedom only for all significant factors and interactions and significance values for both significant and non-significant factors and interactions, for main and interaction terms for each ANOVA type used.
(DOCX)

Table S2 **Results of statistical analysis of firing rate (PFR) and temporal (L_PFR_) measures in single cells responsive to the smooth surface discrimination whisker motion stimulus from 5–30** **ms from stimulus onset (viz.** **Figure 4** **).** Table format as for Table S1.
(DOCX)

Table S3 **Results of statistical analysis of firing rate (PFR) measures in single cells responsive to the rough surface discrimination whisker motion stimulus from 5–30 ms from stimulus onset (viz.** **Figure 4** **).** Table format as for Table S1.
(DOCX)

Table S4 **Results of statistical analysis of Peak Excitatory Firing Rate (PFR) measure in single cells responsive to the first cycle of the free whisking motion stimulus from 5–200** **ms from stimulus onset (viz.** **Figure 5** **).** Table format as for Table S1.
(DOCX)

Table S5 **Results of statistical analysis of firing rate (PFR) and temporal (L_PFR_) measures in single cells responsive to the onset of the free whisking motion stimulus from 5–50** **ms from stimulus onset (viz.** **Figure 5** **).** Table format as for Table S1.
(DOCX)

Table S6 **Results of statistical analysis of firing rate (PFR) and temporal (L_PFR_) measures in single cells responsive to the trapezoidal whisker motion stimulus from 5–50** **ms from stimulus onset (viz.** **Figure 7** **).** Table format as for Table S1.
(DOCX)

Results S1 **Description of patterns of responses in Sham and TBI animals seen in the population Grand PSTHs, to all whisker deflection stimuli.**
(DOCX)
