# Supplementary figures and images for: Sensory Cortex Underpinnings of Traumatic Brain Injury Deficits
Source: PLoS One. 2012 Dec 21;7(12):e52169. doi: 10.1371/journal.pone.0052169 (PMC3528746; doi:10.1371/journal.pone.0052169)

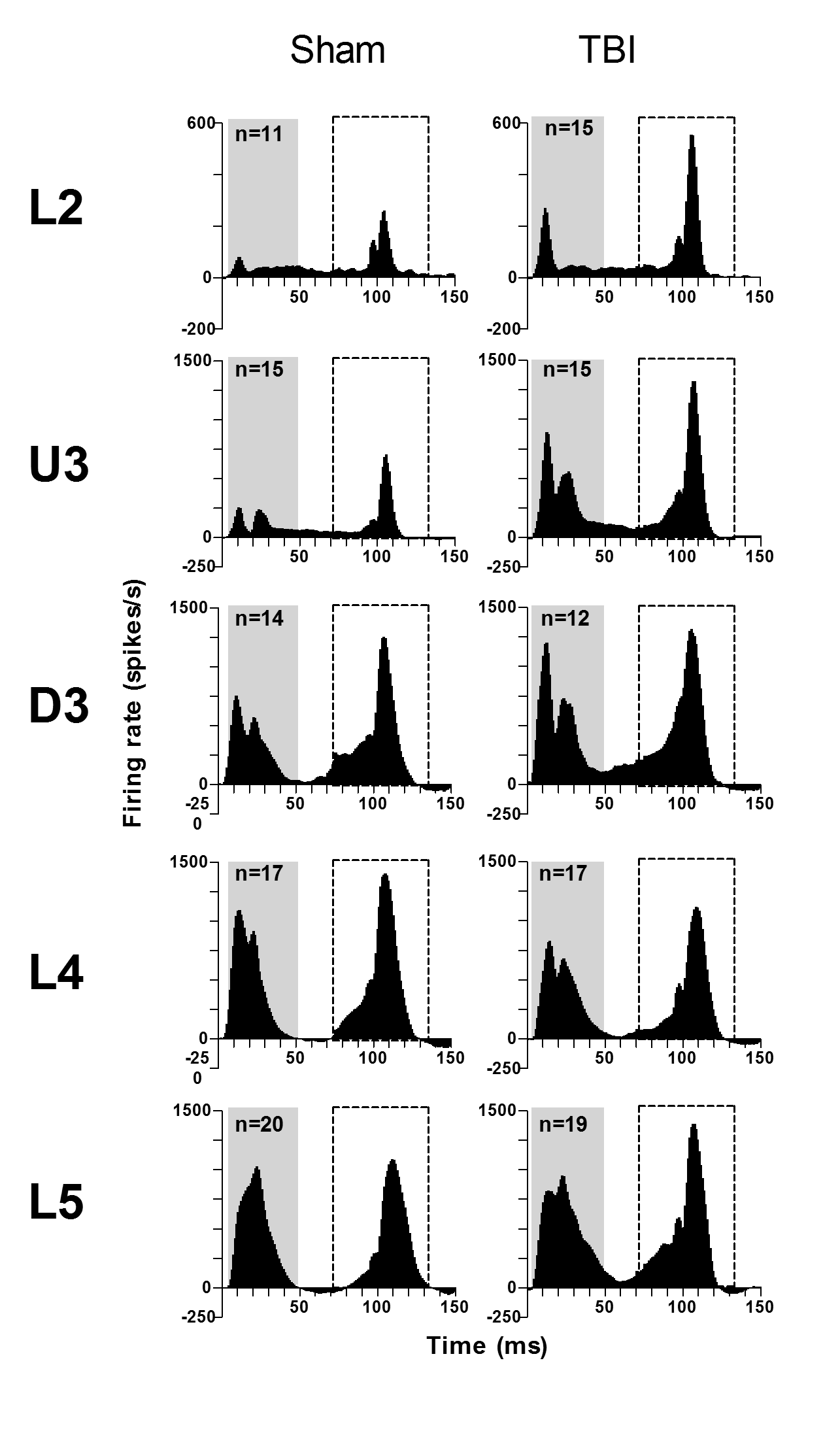

Supplement: Figure S1 — Effects of traumatic brain injury on pattern of responses to the “object contact” whisker motion waveform: Population Grand PSTHs to the largest stimulus waveform (3.6 mm total deflection). Firing rates were averaged across all responsive clusters (firing rates >pre-stimulus spontaneous activity; cluster numbers listed in shaded region) in Sham surgery animals and in TBI animals. The first column of PSTHs is from the Sham surgery cases and the second column from the TBI cases. The boxed areas represent the analysis windows from 5–50 ms post-stimulus onset to capture the onset response (shaded box) and the window from 70–130 ms post- stimulus onset to capture the offset response (dashed box). For all columns the lamina from which data were obtained for each row is indicated to the left of the PSTHs. Cluster numbers for each lamina and group are also presented. (TIF) [file pone.0052169.s001.tif]

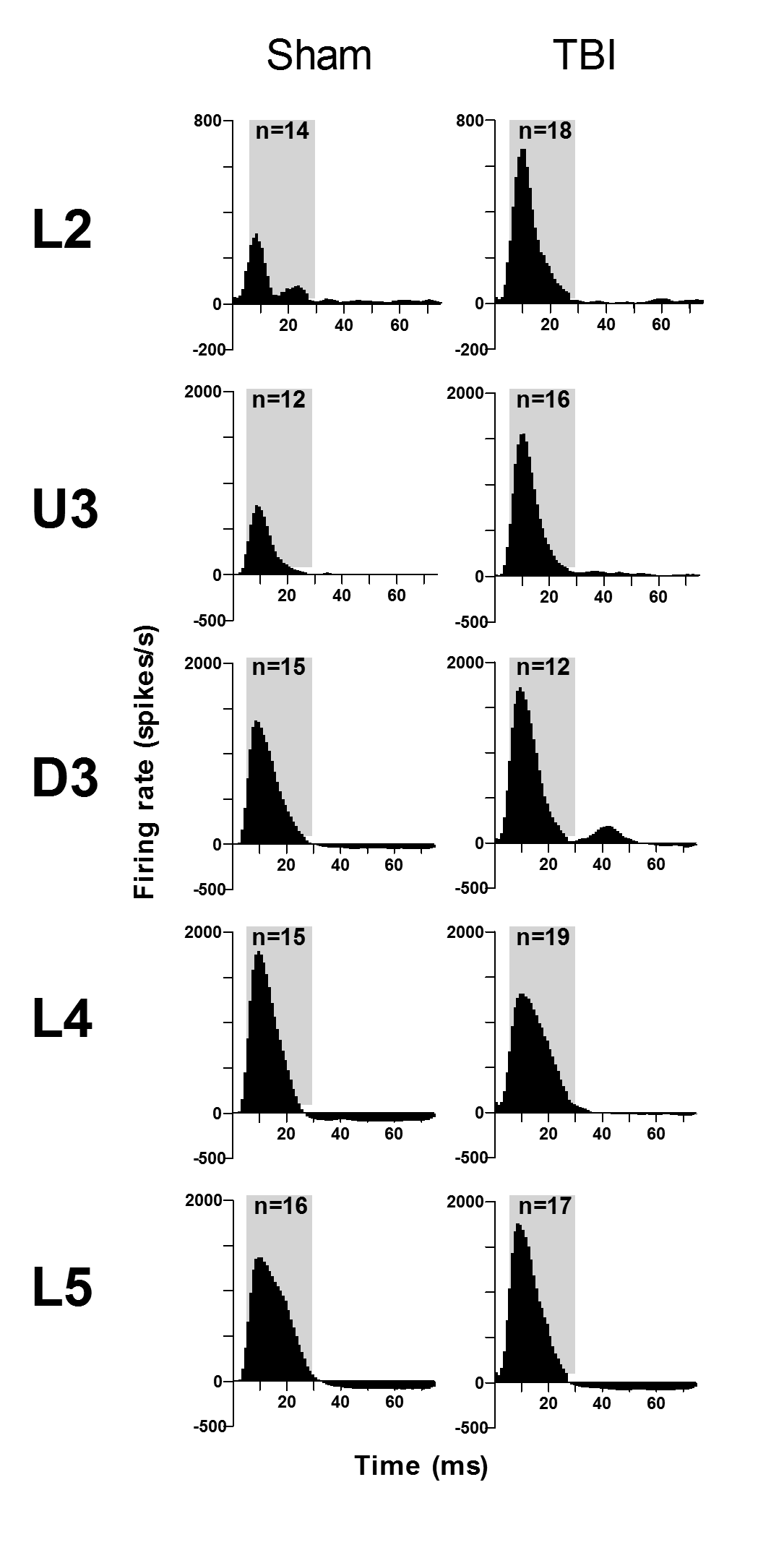

Supplement: Figure S2 — Effects of traumatic brain injury on pattern of responses to the “smooth surface discrimination” whisker motion waveform: Population Grand PSTHs to the largest stimulus waveform (3.6 mm total deflection). Firing rates were averaged across all responsive clusters (firing rates >pre-stimulus spontaneous activity; cluster numbers listed in shaded region) in Sham surgery animals and in TBI animals. The first column of PSTHs is from the Sham surgery cases and the second column from the TBI cases. The boxed areas represent the analysis window from 5–30 ms post-stimulus onset to capture the onset response. Figure conventions as for Figure S1. (TIF) [file pone.0052169.s002.tif]

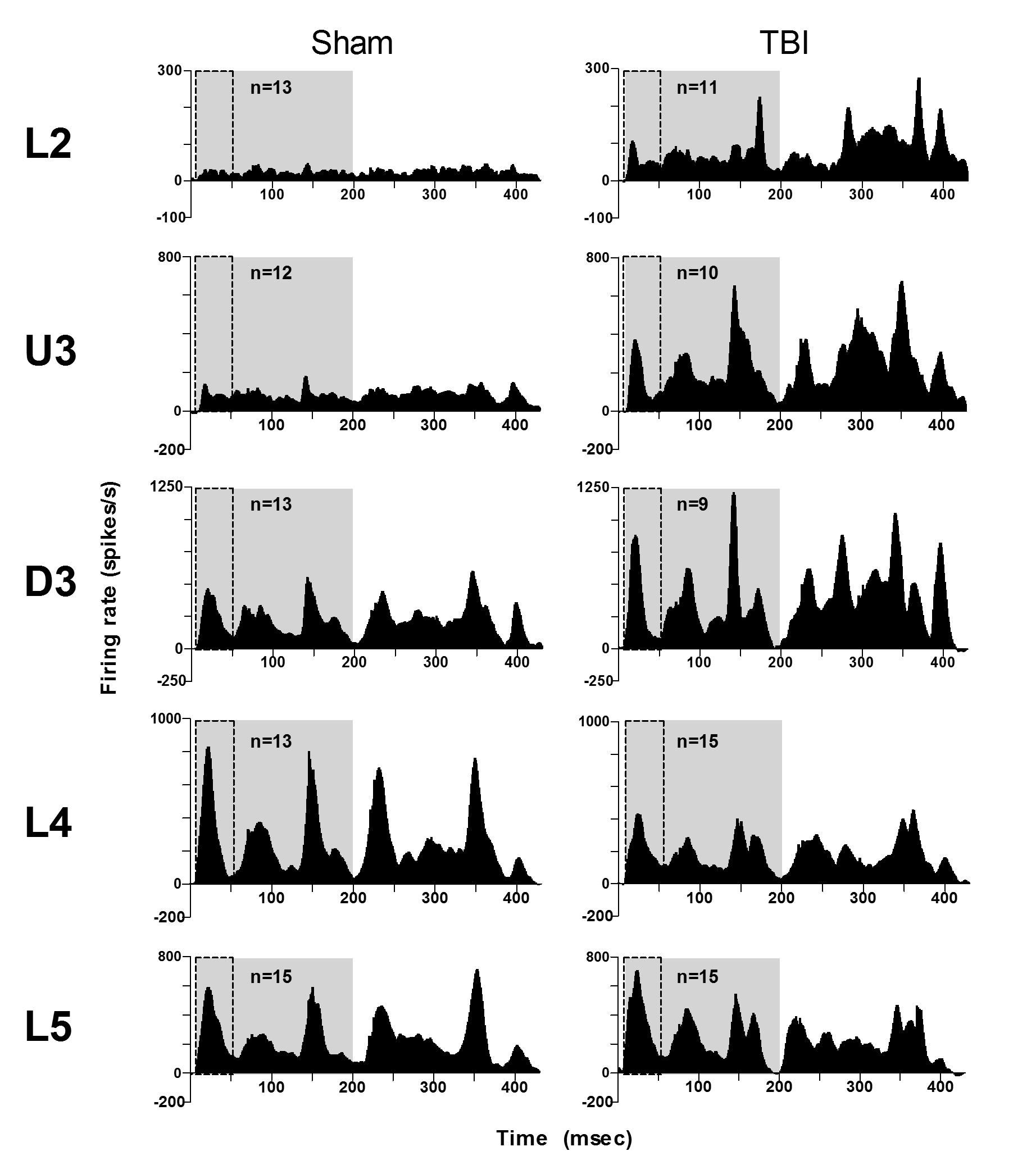

Supplement: Figure S3 — Effects of traumatic brain injury on pattern of responses to the “free whisking” whisker motion waveform: Population Grand PSTHs to the largest stimulus waveform (3.6 mm total deflection). Firing rates were averaged across all responsive clusters (firing rates >pre-stimulus spontaneous activity; cluster numbers listed in shaded region) in Sham surgery animals and in TBI animals. The first column of PSTHs is from the Sham surgery cases and the second column from the TBI cases. The boxed areas represent the analysis windows from 5–50 ms post-stimulus onset to capture the onset response (dashed box) and the window from 5–200 ms post- stimulus onset to capture the first cycle of the stimulus (shaded box). Figure conventions as for Figure S1. (TIF) [file pone.0052169.s003.tif]

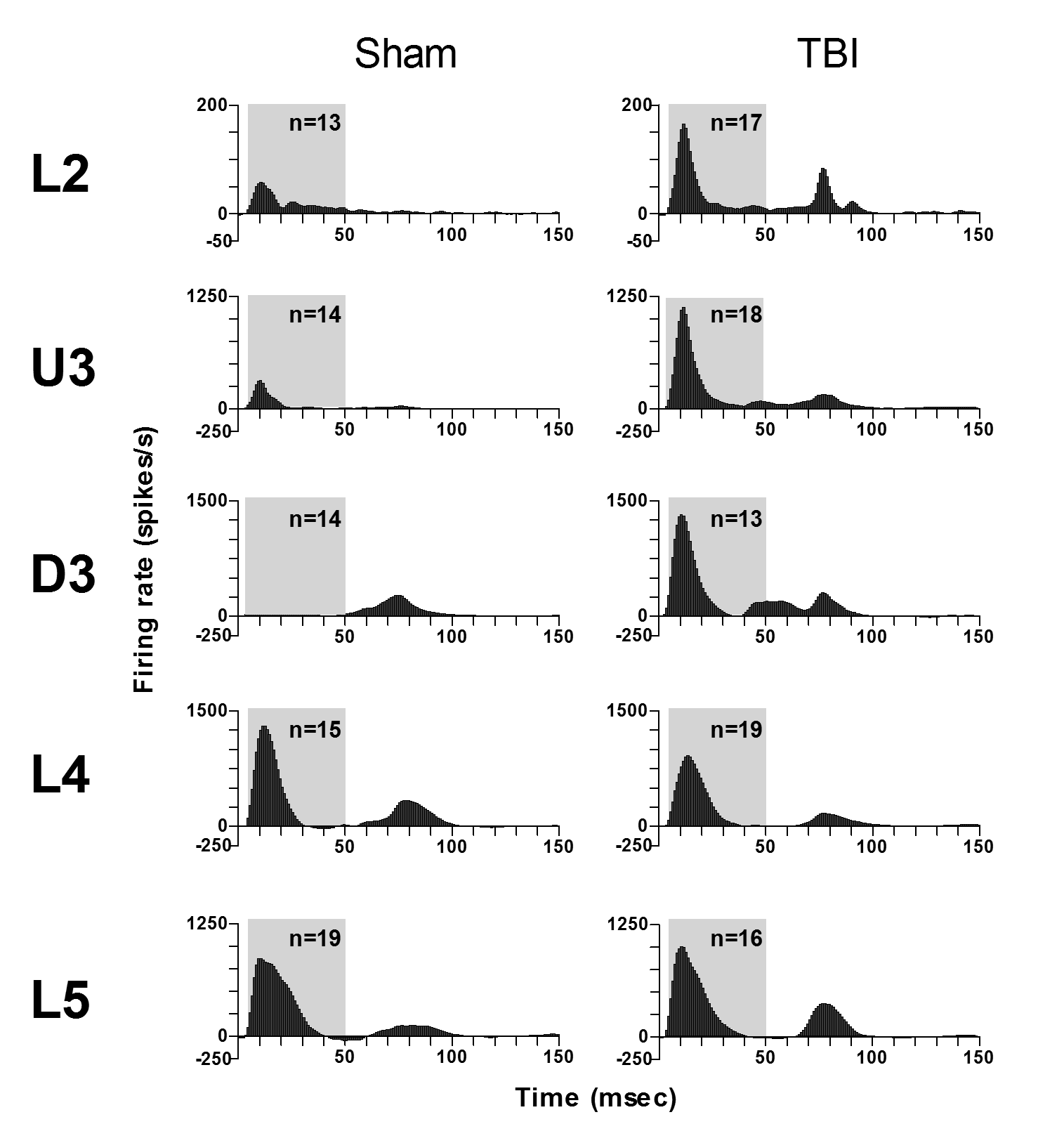

Supplement: Figure S4 — Effects of traumatic brain injury on pattern of responses to the trapezoidal whisker motion waveform: Population Grand PSTHs to the highest stimulus velocity (400 m/s). Firing rates were averaged across all responsive clusters (firing rates >pre-stimulus spontaneous activity; cluster numbers listed in shaded region) in Sham surgery animals and in TBI animals. The first column of PSTHs is from the Sham surgery cases and the second column from the TBI cases. The boxed areas represent the analysis window from 5–50 ms post-stimulus onset to capture the onset response. Figure conventions as for Figure S1. (TIF) [file pone.0052169.s004.tif]
