# Supplementary material for: Sensory Cortex Underpinnings of Traumatic Brain Injury Deficits
Source: PLoS One. 2012 Dec 21;7(12):e52169. doi: 10.1371/journal.pone.0052169 (PMC3528746; doi:10.1371/journal.pone.0052169)
Supplement: Table S1 — Results of statistical analysis of firing rate (PFRon) and temporal measures (LPFR) in single cells responsive to the object contact whisker motion stimulus from 5–50 ms from stimulus onset (related to Figure 3 ). The Table lists F statistics and degrees of freedom only for all significant factors and interactions and significance values for both significant and non-significant factors and interactions, for main and interaction terms for each ANOVA type used. (DOCX) [file pone.0052169.s005.docx]

Supplemental Information for

“Sensory Cortex Underpinnings of Traumatic Brain Injury Deficits”

Dasuni S Alwis, Edwin B Yan, Maria-Cristina Morganti-Kossmann and Ramesh Rajan

^1^Department of Physiology, Monash University, Clayton, VIC 3800, Australia, ^2^National Trauma Research Institute, Alfred Hospital, Prahran, VIC 3004, Australia

Corresponding author:

R Rajan

Department of Physiology,

Monash University, Clayton

VIC 3800

**Tel:** +61 3 990 52525
**Fax:** +61 3 990 52547
**Email:** [Ramesh.Rajan@monash.edu](mailto:Ramesh.Rajan@monash.edu)

This file contains:

**Supplementary Data Table S1*Supplementary Data***

**Table S1. Results of statistical analysis of firing rate (PFR_on_) and temporal measures (L_PFR_) in single cells responsive to the object contact whisker motion stimulus from 5-50ms from stimulus onset (related to Figure 3). The Table lists F statistics and degrees of freedom only for all significant factors and interactions and significance values for both significant and non-significant factors and interactions, for main and interaction terms for each ANOVA type used.**

| Response metric: Peak Excitatory Firing Rate (PFR_on_) in the onset response analysis window from 5-50 ms from stimulus onset**.** | | | | |
| --- | --- | --- | --- | --- |
| **ANOVA type** | **Layer** | **Main terms*** | **Interaction terms*** | |
| ***Mixed-model repeated measures ANOVA (2 Groups x 5 Layers x 10 Amplitudes)*** | All layers | **Group *F* _1,574_ = 6.42, *p* = 0.012**  **Layer *F* _1,574_ = 25.25, p < 0.001**  **Amplitude *F* _4.8,2778_ = 103.05, *p* < 0.001** | **Group x Layer *F* _4,574_ = 6.83, *p* < 0.001**  **Amplitude x Layer *F* _19.4,2778_ = 2.17, *p* = 0.002**  **Amplitude x Group *F* _4.8, 2778_ = 18.09, *p* < 0.001**  Amplitude x Group x Layer *p* = 0.267 | |
| ***Two-way repeated measures ANOVAs (2 Groups x 10 Amplitudes)*** | L2 | **Group *F* _1,70_ = 6.12, *p* = 0.016**  **Amplitude *F* _2,138_ = 12.80, *p* < 0.001** | **Amplitude x Group *F* _2,138_ = 3.44, *p* = 0.036** | |
|  | U3 | **Group *F* _1,97_ = 36.79, *p* < 0.001**  **Amplitude *F* _4.8,462_ = 15.46, *p* < 0.001** | **Amplitude x Group *F* _4.8,462_ = 3.17, *p* = 0.009** | |
|  | D3 | **Group *F* _1,113_ = 5.57, *p* = 0.020**  **Amplitude *F* _4.5,510_ = 24.78, *p* < 0.001** | **Amplitude x Group *F* _4.5,510_ = 6.15, *p* < 0.001** | |
|  | L4 | Group *p* < 0.316  **Amplitude *F* _4.1,597_ = 36.65, *p* < 0.001** | **Amplitude x Group *F* _4.1,597_ = 9.65, *p* < 0.001** | |
|  | L5 | Group *p* < 0.097  **Amplitude *F* _5,735_ = 42.77, *p* < 0.001** | **Amplitude x Group *F* _5,735_ = 4.55, *p* < 0.001** | |
|  |  | | | |
| Response metric: Latency from stimulus onset to the peak firing rate (L_PFR_) in the onset response analysis window from 5-50 ms from stimulus onset**.** | | | | |
| **ANOVA type** | **Layer** | **Main terms*** | | **Interaction terms*** |
| ***Mixed-model repeated measures ANOVA (2 Groups x 5 Layers x 10 Amplitudes)*** | All layers | Group *p* = 0.823  Layer *p* = 0.205  **Amplitude *F* _7.1,4061_ = 47.47, *p* < 0.001** | | **Group x Layer *F* _4,574_ = 3.27, *p* = 0.011**  **Amplitude x Layer *F* _28.3, 4061_ = 2.97, *p* < 0.001**  Amplitude x Group *p* = 0.067  **Amplitude x Group x Layer *F* _28.3, 4060_ = 1.69, *p* = 0.013** |
| ***Two-way repeated measures ANOVAs (2 Groups x 10 Amplitudes)*** | L2 | **Group *F* _1,70_ = 4.83, *p* = 0.031**  **Amplitude *F* _7.2,507_ = 2.17, *p* = 0.034** | | Amplitude x Group *p* = 0.225 |
|  | U3 | Group *p* = 0.663  **Amplitude *F* _7,676_ = 7.86, *p* < 0.001** | | Amplitude x Group *p* = 0.431 |
|  | D3 | Group *F* _1,113_ = 0.99, *p* = 0.323  **Amplitude *F* _5.6,632_ = 21.60, *p* < 0.001** | | Amplitude x Group *p* = 0.066 |
|  | L4 | **Group *F* _1,146_ = 7.51, *p* = 0.007**  **Amplitude *F* _5.6,820_ = 21.65, *p* < 0.001** | | Amplitude x Group *p* = 0.188 |
|  | L5 | Group *p* = 0.254  **Amplitude *F* _6.2,922_ = 32.89, *p* < 0.001** | | **Amplitude x Group *F* _6.2,922_ = 2.13, *p* = 0.045** |

* Greenhouse-Geisser corrections applied where required
