# Supplementary material for: Sensory Cortex Underpinnings of Traumatic Brain Injury Deficits
Source: PLoS One. 2012 Dec 21;7(12):e52169. doi: 10.1371/journal.pone.0052169 (PMC3528746; doi:10.1371/journal.pone.0052169)
Supplement: Table S3 — Results of statistical analysis of firing rate (PFR) measures in single cells responsive to the rough surface discrimination whisker motion stimulus from 5–30 ms from stimulus onset (viz. Figure 4 ). Table format as for Table S1. (DOCX) [file pone.0052169.s007.docx]

Supplemental Information for

“Sensory Cortex Underpinnings of Traumatic Brain Injury Deficits”

Dasuni S Alwis, Edwin B Yan, Maria-Cristina Morganti-Kossmann and Ramesh Rajan

^1^Department of Physiology, Monash University, Clayton, VIC 3800, Australia, ^2^National Trauma Research Institute, Alfred Hospital, Prahran, VIC 3004, Australia

Corresponding author:

R Rajan

Department of Physiology,

Monash University, Clayton

VIC 3800

**Tel:** +61 3 990 52525
**Fax:** +61 3 990 52547
**Email:** [Ramesh.Rajan@monash.edu](mailto:Ramesh.Rajan@monash.edu)

This file includes:

**Supplementary Data Table S3**

***Supplementary Data***

**Table S3. Results of statistical analysis of firing rate (PFR) measures in single cells responsive to the rough surface discrimination whisker motion stimulus from 5-30ms from stimulus onset (viz. Figure 4). Table format as for Table S1.**

| Response metric: Peak Excitatory Firing Rate (PFR) in the onset response analysis window from 5-30 ms from stimulus onset**.** | | | |
| --- | --- | --- | --- |
| **ANOVA type** | **Layer** | **Main terms*** | **Interaction terms*** |
| ***Mixed-model repeated measures ANOVA (2 Groups x 5 Layers x 10 Amplitudes)*** | All layers | **Group *F* _1,532_ = 5.52, p = 0.019**  **Layer *F* _4,532_ = 20.27, p < 0.001**  **Amplitude *F* _4.4,2347_ = 53.01, *p* < 0.001** | Group x Layer *p* = 0.054  **Amplitude x Layer *F* _17.7, 2347_ = 1.82, *p* = 0.019**  Amplitude x Group *p* = 0.148  **Amplitude x Group x Layer *F* _17.7,2347_ = 2.08, *p* = 0.005** |
| ***Two-way repeated measures ANOVAs (2 Groups x 10 Amplitudes)*** | L2 | **Group *F* _1,74_ = 11.87, *p* = 0.001**  **Amplitude *F* _4.1,305_ = 9.29, *p* < 0.001** | **Amplitude x Group *F* _4.1,305_= 3.62, *p* = 0.006** |
|  | U3 | **Group *F* _1,87_ = 9.04, *p* = 0.003**  **Amplitude *F* _3.6,312_ = 16.90, *p* < 0.001** | Amplitude x Group *p* = 0.500 |
|  | D3 | Group *p* = 0.828  **Amplitude *F* _4.5,467_ = 4.89, *p* < 0.001** | Amplitude x Group *p* = 0.314 |
|  | L4 | Group *p* = 0.314  **Amplitude *F* _4.6,608_ = 12.06, *p* < 0.001** | Amplitude x Group *p* = 0.180 |
|  | L5 | Group *p* = 0.236  **Amplitude *F* _4,543_ = 21.61, *p* < 0.001** | **Amplitude x Group *F* _4,543_ = 4.14, *p* = 0.003** |

* Greenhouse-Geisser corrections applied where required
