# Supplementary material for: Sensory Cortex Underpinnings of Traumatic Brain Injury Deficits
Source: PLoS One. 2012 Dec 21;7(12):e52169. doi: 10.1371/journal.pone.0052169 (PMC3528746; doi:10.1371/journal.pone.0052169)
Supplement: Table S6 — Results of statistical analysis of firing rate (PFR) and temporal (LPFR) measures in single cells responsive to the trapezoidal whisker motion stimulus from 5–50 ms from stimulus onset (viz. Figure 7 ). Table format as for Table S1. (DOCX) [file pone.0052169.s010.docx]

Supplemental Information for

“Sensory Cortex Underpinnings of Traumatic Brain Injury Deficits”

Dasuni S Alwis, Edwin B Yan, Maria-Cristina Morganti-Kossmann and Ramesh Rajan

^1^Department of Physiology, Monash University, Clayton, VIC 3800, Australia, ^2^National Trauma Research Institute, Alfred Hospital, Prahran, VIC 3004, Australia

Corresponding author:

R Rajan

Department of Physiology,

Monash University, Clayton

VIC 3800

**Tel:** +61 3 990 52525
**Fax:** +61 3 990 52547
**Email:** [Ramesh.Rajan@monash.edu](mailto:Ramesh.Rajan@monash.edu)

This file contains:

**Supplementary Data Table S6**

***Supplementary Data***

**Table S6. Results of statistical analysis of firing rate (PFR) and temporal (**L_PFR_**) measures in single cells responsive to the trapezoidal whisker motion stimulus from 5-50ms from stimulus onset (viz. Figure 7). Table format as for Table S1.**

| Response metric: Peak Excitatory Firing Rate (PFR) in the onset response analysis window from 5-50 ms from stimulus onset**.** | | | |
| --- | --- | --- | --- |
| **ANOVA type** | **Layer** | **Main terms*** | **Interaction terms*** |
| ***Mixed-model repeated measures ANOVA (2 Groups x 5 Layers x 3 Velocities)*** | All layers | **Group *F* _1,573_ = 4.84, p = 0.028**  **Layer *F* _4,573_ = 10.37, p < 0.001**  **Velocity *F* _1.5,836_ = 171.57, *p* < 0.001** | **Group x Layer *F* _4,573_ = 3.00, *p* = 0.018**  **Velocity x Layer *F* _5.8, 836_ = 8.58, *p* < 0.001**  **Velocity x Group *F* _1.5, 836_ = 6.35, *p* = 0.005**  Velocity x Group x Layer *p* = 0.532 |
| ***Two-way repeated measures ANOVAs (2 Groups x 10 Amplitudes)*** | L2 | Group *F* _1,65_ = 2.85, *p* = 0.096  **Velocity *F* _1.5,97_ = 17.71, *p* < 0.001** | Velocity x Group *p* = 0.083 |
|  | U3 | Group *F* _1,103_ = 2.88, *p* = 0.093  **Velocity *F* _1.2,130_ = 28.46, *p* < 0.001** | Velocity x Group *p* = 0.134 |
|  | D3 | **Group *F* _1,106_ = 9.16, *p* = 0.003**  **Velocity *F* _1.5,161_ = 76.73, *p* < 0.001** | Velocity x Group *p* = 0.080 |
|  | L4 | Group *F* _1,154_ = 1.06, *p* = 0.306  **Velocity *F* _1.4,209_ = 45.86, *p* < 0.001** | Velocity x Group *p* = 0.126 |
|  | L5 | Group *F* _1,145_ = 0.22, *p* = 0.641  **Velocity *F* _1.6,230_ = 51.8, *p* < 0.001** | Velocity x Group *p* = 0.390 |
|  | | | |
| Response metric: Latency from stimulus onset to the peak firing rate (L_PFR_) in the onset response analysis window from 5-50 ms from stimulus onset**.** | | | |
| **ANOVA type** | **Layer** | **Main terms*** | **Interaction terms*** |
| ***Mixed-model repeated measures ANOVA (2 Groups x 5 Layers x 10 Amplitudes)*** | All layers | **Group *F* _1,573_ = 7.08, *p* = 0.008**  **Layer *F* _4,573_ = 6.86, *p* < 0.001**  **Velocity *F* _1.9,1079_ = 63.63, *p* < 0.001** | **Group x Layer *F* _4,573_ = 7.81, *p* < 0.001**  **Velocity x Layer *F* _7.5, 1079_ = 2.21, *p* = 0.027**  Velocity x Group *F* _1.9, 1079_ = 1.30, *p* = 0.271  Velocity x Group x Layer *F* _7.5, 1079_ = 1.61, *p* = 0.122 |
| ***Two-way repeated measures ANOVAs (2 Groups x 10 Amplitudes)*** | L2 | **Group *F* _1,65_ = 12.83, *p* = 0.001**  Velocity *F* _2,127_ = 2.06, *p* = 0.132 | Velocity x Group *F* _2,127_ = 0.43, *p* = 0.649 |
|  | U3 | Group *F* _1,103_ = 0.007, *p* = 0.933  **Velocity *F* _1.9,193_ = 15.96, *p* < 0.001** | Velocity x Group *F* _1.9,193_ = 1.91, *p* = 0.154 |
|  | D3 | **Group *F* _1,106_ = 4.10, *p* = 0.046**  **Velocity *F* _1.8,186_ = 18.52, *p* < 0.001** | **Velocity x Group *F* _1.8,186_ = 4.66, *p* = 0.014** |
|  | L4 | **Group *F* _1,154_ = 7.10, *p* = 0.009**  **Velocity *F* _1.9,284_ = 29.44, *p* < 0.001** | Velocity x Group *F* _1.9,284_ = 1.12, *p* = 0.324 |
|  | L5 | Group *F* _1,145_ = 0.07, *p* = 0.80  **Velocity *F* _1.9,278_ = 33.41, *p* < 0.001** | Velocity x Group *F* _1.9,278_ = 0.90, *p* = 0.402 |

* Greenhouse-Geisser corrections applied where required
