# Supplementary material for: Sensory Cortex Underpinnings of Traumatic Brain Injury Deficits
Source: PLoS One. 2012 Dec 21;7(12):e52169. doi: 10.1371/journal.pone.0052169 (PMC3528746; doi:10.1371/journal.pone.0052169)
Supplement: Results S1 — Description of patterns of responses in Sham and TBI animals seen in the population Grand PSTHs, to all whisker deflection stimuli. (DOCX) [file pone.0052169.s011.docx]

Supplemental Information for

“Sensory Cortex Underpinnings of Traumatic Brain Injury Deficits”

Dasuni S Alwis, Edwin B Yan, Maria-Cristina Morganti-Kossmann and Ramesh Rajan

^1^Department of Physiology, Monash University, Clayton, VIC 3800, Australia, ^2^National Trauma Research Institute, Alfred Hospital, Prahran, VIC 3004, Australia

Corresponding author:

R Rajan

Department of Physiology,

Monash University, Clayton

VIC 3800

**Tel:** +61 3 990 52525
**Fax:** +61 3 990 52547
**Email:** [Ramesh.Rajan@monash.edu](mailto:Ramesh.Rajan@monash.edu)

This file includes:

**Supplementary Results**

***Supplementary Results:*** ***Pattern of responses in Sham and TBI animals seen in the population Grand PSTHs***

***1. Response patterns for Object contact stimulus (see Fig. S1)***

In Sham surgery animals, the population Grand PSTH had two onset peaks (shaded box in Fig. S1) which were clearly separable in all layers except in L5. These were followed, in peri-stimulus time, by low level tonic excitation (in supragranular layers, L2, U3 and D3) or inhibition (L4 and L5). At stimulus offset (dashed box in Fig. S1) there was a set of excitatory peaks which tailed off, in the three deeper layers (D3-L5), into offset inhibition. The same general pattern of activity was seen in the TBI cases but a striking difference was a marked *increase* in excitation in onset and offset responses in all supragranular layers (L2, U3 and D3). However, in L4 there was *reduced* excitation in onset and offset responses and in peri-stimulus inhibition between these two components, but not in offset inhibition. In L5 in TBI cases there was a weaker but broader peak onset excitation and an increase in offset excitation; there was also a reduction in peri-stimulus inhibition between onset and offset excitation peaks, but not in offset inhibition. In general, compared to cells in Sham surgery animals, TBI cells showed an increase in excitation in all supragranular layers for onset and offset responses, a decrease in excitation in the granular layer for onset and offset responses, and relatively minor changes in the infra-granular layer.

***2. Response patterns for Smooth surface discrimination stimulus (see Fig. S2)***

This stimulus contained about 7 oscillatory motions of the whiskers (see Fig. 1). The Grand PSTHs generated from all responsive multi-unit clusters to this stimulus showed that across all layers the dominant response element to was a single onset excitatory peak sometimes followed (especially in supra-granular layers) by a small second peak within (Sham L2) or outside (TBI D3) the stimulus period (Fig. S2). Generally, very similar – if not identical – response patterns were seen in all layers in the two groups, with some evidence for a change in post-stimulus response patterns in U3 and D3; in U3 there was post-stimulus tonic excitation in TBI cases but not in Sham surgery cases and in D3 there was a post-offset inhibition in Sham surgery animals and a post-offset excitation in TBI cases. In L4 and L5 there was evidence for post-stimulus inhibition in both groups, more strikingly so in the Sham surgery cases but also, to a smaller extent, in the TBI cases, suggesting that the loss of inhibition was restricted to upper layers L2 and U3. Overall, TBI cells showed similar response patterns to those seen in Sham surgery cells, except for a post-offset excitation in U3 and D3, with evidence of loss of post-offset inhibition in D3.

***3. Response patterns for Rough surface discrimination stimulus (see Fig. 6)***

The Grand PSTHs to this stimulus were generally similar to those seen with the “smooth surface discrimination” waveform described above (Fig. S2). As in that case, across all layers the dominant response element was a single onset excitatory peak which was followed by some small tonic post-stimulus excitation in the top two layers and by post-stimulus inhibition in all other layers. Other than some longer-lasting tonic activity in L2 and U3, especially post-stimulus responses, nearly identical response patterns were seen in all other layers in the two groups, including post-stimulus inhibition in D3, L4 and L5.

***4. Response patterns for exploratory whisking stimulus (see Fig. S3)***

In the population of responsive multi-unit clusters, this stimulus generated a number of complex response components in both experimental groups. Responses could be separated into two blocks each aligned with one of the two cycles of the motion waveform. For brevity of description we will describe the response components seen in the period from stimulus onset to 200msec post-stimulus onset (shaded box in Fig. S3), the period aligned with the first cycle of the stimulus, since the response components aligned with the second cycle of the stimulus were generally similar.

In Sham surgery animals, response patterns to this first cycle (and to the other cycle) were not well defined in the two uppermost layers (L2 and U3) but were well defined in the three deeper layers (D3, L4, and L5). In the latter layers the Grand PSTH consisted of three clear peaks of excitation to the first cycle of whisker motion. One peak was aligned to the stimulus onset and the up-ramp of the first cycle, a second excitatory response peak was aligned to the peak of this cycle, and the third excitatory response peak aligned to the down-ramp of this cycle. Response patterns to the second cycle of whisker motion consisted of similar excitatory response peaks though sometimes not as clearly separated as for the first cycle (see, e.g., L4 responses). This second cycle of responses was followed, in all layers except L2, by a small post-stimulus offset excitatory response.

Overall, similar response patterns were generally seen in all layers in the two groups. However TBI cells showed laminar-dependent changes in response strength compared to Sham surgery cases. In all supra-granular layers L2, U3 and D3 and the different response peaks which were poorly or not at all defined in L2 and U3 in Sham surgery cases were much better defined in the TBI cases. This was true of responses to both stimulus cycles and in the post-stimulus offset response – in these supra-granular layers, response strength to the second cycle could be stronger both in the peaks and in the overall responses. A second notable effect was the contrast between these changes in the supra-granular layers and the change seen in the granular layer: response strength to both stimulus cycles were clearly *reduced* in TBI cases compared to Sham surgery cases. Finally, in the infra-granular layer, there was a small reduction in response strength to both stimulus cycles in TBI cases compared to Sham surgery cases. Note that in both groups there was no instance of inhibition (a reduction in responses below the y = 0 Hz firing rate line), a feature seen for all of the other three naturalistic motion waveforms.

***5. Response patterns for trapezoidal stimuli (see Fig. S4)***

Only the Grand PSTHs to the trapezoid with the fastest onset ramp velocity (400 mm/sec) are presented, to exemplify the pattern of responses to this whisker motion pattern. The pattern of responses was very similar between the two experimental groups with the major feature across all layers being an early excitatory response, at ~10 msec post stimulus onset, which increased in strength with increasing ramp velocity (velocity changes not shown). At the highest velocity, at which strong onset responses were elicited in all layers, this response was most tightly focused temporally in L2, U3 and L4, a bit more dispersed in D3 and very much broader in L5. In some layers there were also later excitatory components. In D2 there was a peri-stimulus tonic excitation which, in TBI cases was followed by an offset response. The same pattern was seen in U3 in TBI cases but not in Sham surgery cases. In D3, L4 and L5, the pattern of responses was remarkably similar in both groups with a strong offset response. In L4 and L5 the onset and offset responses were separated by a period of low level inhibition in Sham surgery animals but not in TBI cases.

Finally, the offset ramp, which had a fixed velocity regardless of onset ramp velocity, elicited at least one excitation peak in all layers (and, in D3 and L4, a second response too). However, the offset response appeared to depend on response strength to the onset ramp: as onset ramp velocity increased (not shown) and correspondingly onset response strength, the strength of the offset response declined, suggesting adaptation effects due to the increasing strength of the onset ramp response.

Thus, in general, response patterns were similar in the two groups except for a stronger offset response in the top two layers in TBI cases compared to Sham surgery cases and an apparent absence of peri-stimulus inhibition in granular and infra-granular layers in the TBI animals.
